# Trace Elements in Edible Flowers from Italy: Further Insights into Health Benefits and Risks to Consumers

**DOI:** 10.3390/molecules25122891

**Published:** 2020-06-23

**Authors:** Giuliana Drava, Valeria Iobbi, Rafaël Govaerts, Vincenzo Minganti, Andrea Copetta, Barbara Ruffoni, Angela Bisio

**Affiliations:** 1 Dipartimento di Farmacia, Università degli Studi di Genova, Viale Cembrano 4, 16148 Genova, Italy; valeria.iobbi@edu.unige.it (V.I.); minganti@difar.unige.it (V.M.); bisio@difar.unige.it (A.B.); 2 Biodiversity Informatics and Spatial Analysis, Royal Botanic Gardens, Kew Jodrell Laboratory, Kew Road, Richmond TW9 3DS, UK; R.Govaerts@kew.org; 3 CREA Centro di Ricerca Orticoltura e Florovivaismo, Corso Inglesi 508, 18038 Sanremo, IM, Italy; andrea.copetta@crea.gov.it (A.C.); barbara.ruffoni@crea.gov.it (B.R.)

**Keywords:** essential elements, ICP OES, metals, mineral composition, toxic elements

## Abstract

The use of edible flowers in cooking dates back to ancient times, but recently it is gaining success among the consumers, increasingly attentive to healthy and sustainable foods of high quality, without neglecting taste, flavour, and visual appeal. The present study aims to deepen the knowledge regarding the mineral composition of edible flowers, an aspect not widely investigated in scientific literature. The concentrations of Cd, Co, Cu, Fe, Mn, Ni, Pb, Sr, V, and Zn have been determined by Inductively Coupled Plasma Optical Emission Spectrometry (ICP OES) in flowers belonging to a wide variety of species. The study highlights that some floral species are characterized by significantly higher concentrations of certain trace elements, e.g., the flowers of *Acmella oleracea* for Mn, those of basil (*Ocimum basilicum*) and of pumpkins (*Cucurbita moschata* and *C. pepo*) for Cu and Sr, and those of orange daylily (*Hemerocallis fulva*) for Ni. Potentially toxic elements are present at low concentrations, often below the limit of the detection for Cd, Co, Ni, V. In all samples, Cd and Pb are well below the maximum permitted levels in foodstuffs. It can be concluded that the edible flowers analyzed can be considered a good source of essential elements and do not present risks for the consumer health as for the mineral composition.

## 1. Introduction

The term “edible flowers” includes a large number of floral species and varieties which have been used for centuries in many parts of the world, both as ingredients of food recipes and as a decoration, especially for “celebrative” preparations. Although edible flowers can be still considered as a niche market, recently they have become increasingly popular [1,2], because adding flowers to dishes can be a good way to enrich taste, flavor, texture and visual aspect of food [3,4]. Depending on the species, the whole flowers, especially the small ones, or only some parts, in the case of medium and large flowers, are consumed in various ways, namely fresh, dried, candied, or differently cooked. They are mainly used in salads, teas, jams, as a garnish mostly for desserts or cocktails, but creative use has no limits, considering the variety of their sensory properties, including shape and color.

A high number of species of edible flowers, around 180 according to Lu et al. [5], are known worldwide. Some of them can be considered as widely consumed vegetables: it is the case of artichokes, zucchini flowers, cauliflowers and saffron. Others are less common as the presence of some species is restricted to certain geo-climatic areas (e.g., hibiscus, mint, sage, pansy, basil), therefore their use is typical of regional cuisines.

From a nutritional point of view, edible flowers are very low in fat and rich in nutrients such as proteins, minerals, vitamins, and bioactive compounds, as flavonoids, carotenoids and anthocyanins that determine their color [1,4,5,6]. Most of the studies dedicated to edible flowers are focused on nutritional aspects and on health benefits, especially related to antioxidant and antimicrobial activities [7,8], while a few papers deal with the content of trace elements [9,10,11,12,13], despite their relevance for human health.

The presence of trace elements in plants may be related to several factors, such as soil composition, watering and atmospheric pollution, and is strongly depending on the plant species. However, the plant parts usually investigated are roots and leaves, very rarely flowers. Actually, hundreds of plants are known as metal hyper-accumulators [14,15], being able to tolerate one or more trace elements [16,17] even at high levels, concentrating them in their tissues without relevant toxic effects. It has also been hypothesized that hyperaccumulation might play a role as a defensive strategy, contributing to protect plants from pathogens, insects and herbivores [18,19,20]. Besides elements whose toxicity is well-known, such as cadmium and lead, the concentrations of several micronutrients (e.g., cobalt, copper, iron, manganese, nickel, zinc), which are necessary for plant growth and development, have to be measured. In fact, these elements, although essential also for human health, may be toxic or may induce allergic reactions when beyond certain values [13,21].

The aim of the present work is to provide a further insight into the chemical composition of edible flowers for a more complete definition of the nutritional profile and safety of use. The concentrations of 10 trace elements (Cd, Co, Cu, Fe, Mn, Ni, Pb, Sr, V, and Zn) were determined by Inductively Coupled Plasma Optical Emission Spectrometry (ICP-OES) in samples of edible flowers from the Ligurian Riviera (North-Western Italy) belonging to 29 species, a wide choice including not only those traditionally used in cooking, but also species mainly known as ornamentals and only recently employed as food. For each species an extensive study of the main botanical characteristics was also performed. The measured concentrations were compared with literature data, although the data available are still scarce and referred to a limited number of floral species and of chemical elements.

## 2. Results

The floral species analyzed are listed in Table S1 together with their main botanical characteristics and the related references therein [7,22,23,24,25,26,27,28,29,30,31,32,33,34,35,36,37,38,39,40,41,42,43,44,45,46,47,48,49,50,51,52,53,54,55,56,57,58,59,60,61,62,63,64,65,66,67,68,69,70,71,72,73,74,75,76,77,78,79,80,81,82,83,84,85,86,87,88,89,90,91]. Table 1 reports the concentrations of the 10 trace elements, expressed as µg/g dry weight: for each element in each floral species, means and standard deviations were computed after analysis of a number from 2 to 6 of composite samples, obtained as described in Section 4.2.

In most cases, low concentrations were measured; in particular, 68% of the measurements were below the detection limit for Cd (0.020 µg/g dry weight), 26% for Co (0.030 µg/g dry weight), 19% for V (0.020 µg/g dry weight), and 10% for Ni (0.070 µg/g dry weight).

**Figure 1:**
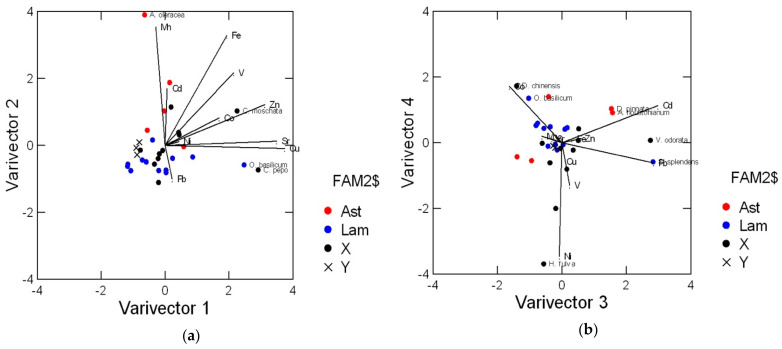
Results of Principal Component Analysis after Varimax rotation, performed on the data matrix of 29 floral species belonging to different botanical families (• Asteraceae; • Lamiaceae; • other) described by 10 trace elements. The x symbols indicate snapdragon flowers (*Anthirrinum majus*) of different colors. Only the names of species at the highest element concentrations are reported. (**a**) Biplot of varivectors 1 and 2; (**b**) Biplot of varivectors 3 and 4.

Principal component analysis [92] was used as an unsupervised pattern recognition method, allowing to extract and visualize the information contained in the data set (i.e., grouping of samples, presence of outliers, correlated variables, relationships between samples and variables): the first two Components retained the 51% of the total variance, and 75% was explained by the first four. Varimax rotation of loadings was performed in order to obtain an easier interpretation of the results. Figure 1a reports the biplot of samples and variables on the plane of the first two varivectors. Most of the samples were grouped at similar, low element concentrations; only the flowers of basil (*Ocimum basilicum*) and of pumpkins (*Cucurbita moschata* and *C. pepo*) showed high concentrations of Cu and Sr on varivector 1 (3 times the mean value for the other species), while the flowers of *Acmella oleracea* were well separated on varivector 2 at high content of Mn (more than 10 times the mean value for the other species).

In Figure 1b the biplot of varivectors 3 and 4 showed as different from the majority of the samples the flowers of scarlet sage (*Salvia splendens,* 3 times the mean Pb concentration), flossflower, dahlia and violet (*Ageratum houstonianum*, *Dahlia pinnata* and *Viola odorata*, 3 times the mean Cd concentration), dianthus and basil (*Dianthus chinensis* and *O. basilicum*, 4 times the mean Co concentration), separated on varivector 3: the flowers of orange daylily (*Hemerocallis fulva*, 6 times the mean Ni concentration) were separated on varivector 4.

Significant correlations between Cu and Sr concentrations (*r* = 0.741, *p* = 0.000) and between Fe and V (*r* = 0.688, *p* = 0.000) were found.

In the case of snapdragon flowers (*Antirrhinum majus*) samples of white, yellow and orange petals were analyzed separately, considering that color may be a relevant factor influencing not only the consumer acceptability of edible flowers but also their chemical composition. As shown in Figure 1, the concentrations of trace elements in the three samples were similar, thus the mean values were used in Table 1.

Moreover, the differences among botanical families were investigated, although this study was possible only for Asteraceae and Lamiaceae, being the only families represented by more than two species (respectively, 5 and 12). As shown in Figure 1a, the flowers of Asteraceae were characterized by higher concentrations of Cd, Fe and Mn: these differences were found significant on the basis of Mann-Whitney test (*p* < 0.01).

## 3. Discussion

Considering that the flowers of the present study were organically grown in the same place, following the same agricultural practice, the differences detected in their mineral composition can be related to the species. Anyway, even when higher contents of certain trace elements were measured, as highlighted by PCA, the concentrations were far lower than those present in hyperaccumulator species.

Unlike the bioactive compounds responsible for antioxidant activities (flavonoids, xanthophylls and carotenoids), which content is related to petal colours [93], no difference was found among the concentrations of trace elements in flowers of different colors belonging to the same species.

Several Authors highlighted the lack of chemical and toxicological data regarding edible flowers, both spontaneous and cultivated [4,21]. The large number of data here collected contributes to increasing the knowledge in a domain scarcely explored in literature. In fact, the studies regarding trace elements in plants are mainly focused on edible and medicinal herbs, either wild or cultivated, on grasses used as pastures or plants used as bioindicators of metal pollution or bioremediation agents, thus the plant parts investigated are in most cases leaves and roots [94,95,96,97,98], while flowers are very rarely studied.

Moreover, the selection of edible flowers may strongly differ from country to country. At our knowledge, no data regarding trace elements are available in literature for many of the species here analyzed. For instance, this is the case of the flowers of *A. houstonianum* having high concentrations of Cd and other elements (Table 1): these data are interesting, considering that this plant had shown a remarkable ability to accumulate trace elements from the soil and to translocate them from roots to shoots, being a good candidate for the phytoremediation of soil contaminated by metals, especially Pb, in a study performed in a mine area in Vietnam [99]. In our study, high concentrations of several elements, in particular Cd, Co, Mn, Ni, and Pb, were measured in the flowers of *Begonia cucullata* var. *cucullata*, *Tagetes erecta* and *S. splendens*: it has to be noted that Ding and Hu [100], in their study on the potential of ornamental plants as bioindicators in urban environments, had recommended these three species on the basis of the levels of metals in leaves and roots. The higher concentrations of Co, Cu, Sr and Zn observed in the flowers of *O. basilicum* can be related to the capacity of accumulating several metals in the shoot and roots of plants grown in natural and contaminated soils [101]. Regarding the flowers of Cucurbitaceae (*C. pepo* and *C. moschata*), no data about high concentrations of Cu and Sr were found in the literature, although the seeds were reported to be rich in minerals (among the others, Cu, Fe, Sr, Zn) [102,103]. Considering that pumpkin flowers are widely consumed, their mineral profile is an added value: moreover, Sotelo et al. [104] reported a high protein content and no antinutritional factors in this floral species.

As for the safety of edible flowers, presently only a few of them (and none of the species of this study) appear on official lists, such as the Novel Food Catalogue [105].

The European Union sets maximum levels for Cd and Pb in foodstuffs; assuming that the chemical composition of edible flowers is not too different from other plant organs [1], it is possible to refer to leaf vegetables, where the maximum levels are 0.20 mg/kg fresh weight for Cd [106] and 0.30 mg/kg fresh weight for Pb [107]. None of the samples showed concentrations beyond these limits. The maximum Cd concentration measured in *D. pinnata* was 0.04 mg/kg fresh weight and the maximum Pb concentration measured in *S. splendens* was 0.16 mg/Kg fresh weight. Moreover, the mean concentrations were much lower.

Regarding the other elements, data are not sufficient to derive a tolerable upper intake level [108], i.e., a maximum level of total chronic intake judged to be unlikely to pose a risk of adverse health effects in humans. Only for Cu (from 1 mg/day for children to 5 mg/day for adults) and for Zn (from 7 to 25 mg/day) this level has been indicated [109], but the small amount of flowers consumed does not pose health risks. Analogously, since an acceptable Mn intake with diet is estimated in the range 1–10 mg/day [109], the use of the spicy flowers of *A. oleracea* in some recipes cannot cause a relevant intake of this metal. The mean dietary intake of V is about of 10-20 µg/person/day, but again this value cannot be exceeded when edible flowers are included in diet. Finally, eating food containing Ni can be responsible for dermal reactions [109] in sensitized individuals, but the edible flowers give a scarce contribution to the dietary intake of this metal.

An extensive comparison between our data and the available literature data was performed (Table 2). All of the cited papers reported data of Cu, Fe, Mn, Zn, while the determination of Cd, Co, Ni, Pb, and Sr was perfomed only in some studies, and no data at all were available for V. In addition, the published studies generally include a small number of floral species. Anyway, the values collected in Table 2 show that the concentrations measured in the present study are generally in agreement with literature data, and in many cases are lower than those reported in other studies, although the comparison is made difficult by the enormous variety of geographical origins (Spain, Portugal, Poland, Czech Republic, Russia, Mali, Burkina Faso) and types of samples (from controlled cultivations or from local markets, from clean or polluted areas).

## 4. Materials and Methods

### 4.1. Plant Materials and Plant Culture

Plant production varied according to species and/or varieties. *A. oleracea, A. houstonianum, A. majus, B. cucullata* var. *cucullata, C. moschata, C. pepo, D. pinnata, D. chinensis, Hibiscus sabdariffa, O. x africanum, O. basilicum, S. farinacea*, the two species of Tagetes (*T. erecta* and *T. lemmonii*) and *V. odorata* were propagated by commercial seed. *Agastache aurantiaca, Fuchsia regia, Monarda didyma, Nepeta x faassenii, S. discolor, S. elegans, S. greggii, S. microphylla, S. splendens, S. x jamensis*, and *Verbena bonariensis* were propagated by cutting. *M. didyma, Nepeta x faassenii and V. bonariensis* were derived from plants purchased at L’Erbaio della Gorra plant nursery (Casalborgone, TO, Italy); *A. aurantiaca, F. regia* were provided by CREAM (Nice, France); sage species and varieties were part of the collection at CREA Institute (Sanremo, IM, Italy). Finally, *H. fulva*, *Tulbaghia cominsii* and *T. violacea* were propagated by division of rhizomes and bulbs, respectively. It must be underlined that the accepetd name for the begonia used in this study is *B. cucullata* var. *cucullata*, unless it is sometimes reported as *Begonia × semperflorens-cultorum* as described by Krauss [113] (*Begonia x semperflorens-cultorum hort.; Begonia Semperflorens-Cultorum Group; Begonia semperflorens-cultiform Hort; Begonia semperflorens-cultorum* H.K.Krauss; *Begonia x hortensis* I.Grafl. & Zwicky).

The seeds of the different species were sown in seedbeds and, after adequate development, the plants were transplanted in pots such as rooted cuttings. Based on the development of plant roots, the pots were 30 cm of diameter with 9 L of volume or 14 cm of diameter with 0.5 L of volume. The pots contained peaty substrate and pumice (70% and 30%, respectively) (Hochmoor Vulcan– Terflor, Capriolo, BS, Italy) with slow release fertilizer (Nitrophoska, Eurochem Agro, Cesano Maderno, MB, Italy). All species were grown at CREA in a greenhouse with anti-insect net, and fertirrigated with nutrient solution (Ferti 3, Planta-Dȕngemittel, Regenstauf, Germany) every week. The plants were grown organically without the use of pesticides as reported in Najar et al. [114]. Briefly, antagonist insects (*Adalia bipunctata*, *Aphidius colemani*, *Amblyseius swirskii*, *Chrysoperla carnea*, *Phytoseiulus persimilis* - Koppert Italia Srl., Bussolengo, VR, Italy) and *Bacillus thuringensis* subsp. Kurstaki (Serbios Srl, Badia Polesine, RO, Italy) were applied in the greenhouse again aphids, thrips, and caterpillars.

### 4.2. Sample Preparation

Fresh flowers at full maturity stage of all species were collected in the morning, between 8 and 10 a.m.; according to the edible use, either the whole flower or only the petals were weighed, packed in paper bags and frozen at –80 °C. Then the samples were freeze dried (Super Modulyo, Edwards, UK) and weighed again in order to compute the ratio between fresh and dry weight. The mean water content found was 81% with data ranging from 74% to 89%.

**Figure 2:**
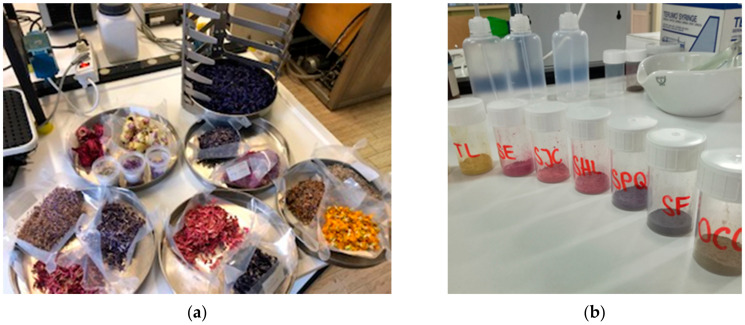
Two of the steps of sample preparation. (**a**) Samples after freeze-drying; (**b**) Samples after homogenization performed in 25 mL Teflon grinding jars with zirconium oxide grinding balls (MM2 Mixer Mill, Retsch, Germany).

After homogenization in 25 mL Teflon grinding jars with zirconium oxide grinding balls (MM2 Mixer Mill, Retsch, Germany), aliquots of 0.13–0.15 g of each floral species were weighed in 120 mL PTFA advanced composite vessels (MDS 2000, CEM Corporation, USA) and mineralized using 5 mL of 65% (m/m) nitric acid (‘for trace analysis’, Scharlau Chemie SA, Spain) in a microwave digestion system (MDS 2000, CEM Corporation, USA) according to the following program: 280 W for 5 min, 440 W for 10 min, and 600 W for 25 min. The solutions, after cooling, were transferred to 25 mL volumetric flasks, diluted to volume using Type I water (>18 MΩ cm) (Puranity TU3+, VWR International bvba, Belgium) and filtered using 0.45 μm Nylon filters (VWR International LLC, USA). For each floral species this procedure allowed to obtain a composite sample, where the number of flowers was dependent on their size (a minimum of 5–6 for the largest flowers, e.g., *C. pepo*). Figure 2 shows two steps of the sample preparation.

### 4.3. Analytical Determinations

The concentrations of Cd, Co, Cu, Fe, Mn, Ni, Pb, Sr, V, and Zn were measured using atomic emission spectrometry with an inductively coupled plasma source (iCAP 7000 Series, Thermo Scientific, UK). An axial plasma view and a desolvation nebulizer (APEX-E with a MicroFlow PFA-ST concentric nebulizer, Elemental Scientific Inc., USA) were employed to achieve greater sensitivity. The instrument was operated following the indications of the manufacturer. The wavelengths used were selected by means of a built-in library: the selection, performed by the Element Finder plug-in of Qtegra Intelligent Scientific Data Solution (ISDS) software 2.7 (Thermo Scientific, UK) was based on high sensitivity, avoiding interferences with the wavelengths of the other elements, including elements present in the samples and potentially interfering, although not measured (e.g., Ca, Mg, Na, P). Plasma was operated at 1150 W RF power, with a gas (Ar) flow of 12 L/min and an auxiliary flow of 0.5 L/min, with Ar in nebulizer at 0.15 MPa.

Calibration was performed with “matrix matching standards” (standard addition method), adding standard solutions in order to match as much as possible the natural concentration of each trace element in the samples to be studied. An addition of 4 µg/mL of Y as internal standard allowed to compensate for matrix difference. All samples were analyzed in duplicate or triplicate and the mean of the results was used. All concentrations were expressed on a dry weight basis. Two blanks were analyzed with each set of samples and subtracted from all measurements. All glassware was washed with 3 M nitric acid and rinsed with Type I water. Wavelengths (nm) and detection limits of the method (based on three times the standard deviation of blanks, as µg/g dry weight) are reported in Table 3.

Accuracy was assessed by analyzing (*n* = 7) the Standard Reference Material NIST1515 (apple leaves, National Institute of Standards and Technology, NIST). Although NIST1515 is a suitable material (same matrix) for accuracy assessment for most of the trace elements considered, in some cases (Cd and Co) the certified concentrations were below the detection limit of the method or too close to it. For this reason, a second certified material with higher concentrations, BCR 482 (lichen *Pseudevernia furfuracea*, European Commission, Joint Research Center – Institute for Reference Materials and Measurements, IRMM), was also used (*n* = 4). The results of the quality control, reported in Table 3, were satisfying: in all cases recoveries were between 85% and 115% (mean recovery 98%).

### 4.4. Data Analysis

Systat software for Windows Version 13 (Systat Software Inc., USA) was used for statistical analysis and to produce graphs. Principal component analysis (PCA) with Varimax rotation was used for explorative data analysis, as unsupervised pattern recognition technique [91]. The correlations between variables were computed using the Pearson correlation coefficients. Mann–Whitney test, as non-parametric analog of the two-sample t-test, was used for comparing the trace element concentrations in samples belonging to different botanical families. Statistical significance was set at *p* value < 0.05.

## 5. Conclusions

The large number of data collected in this study allowed to increase the knowledge in a domain scarcely explored in literature. In fact, the investigation performed covers a wide variety of edible floral species, providing further insights into their health benefits and safety. Moreover, the detailed study of the botanical characteristics of the plant species highlighted their wide geographical diffusion: actually, most of these flowers are consumed in many parts of the world, although they are known under different common names and prepared following a variety of recipes, both traditional and innovative. Regarding the trace elements analyzed, the studied species can be considered a good source of essential elements, without risks for the consumer health. No metal hyperaccumulators were found among the flowers analyzed.

## Figures and Tables

**Table 1:** Concentrations (expressed as μg/g dry weight) of 10 trace elements in edible flowers belonging to 29 species. Data are reported as mean value ± standard deviation. Summary statistics (mean ± standard deviation, median and range) referred to the whole data set are also shown.

Species	Cd	Co	Cu	Fe	Mn	Ni	Pb	Sr	V	Zn
	(µg/g Dry Weight)									
*Acmella oleracea* (L.) R.K.Jansen	0.036 ± 0.014	0.214 ± 0.149	6.17 ± 0.40	122.5 ± 38.0	307.4 ± 169.9	0.60 ± 0.15	0.19 ± 0.13	15.94 ± 1.22	0.119 ± 0.037	38.4 ± 7.7
*Agastache aurantiaca* (A.Gray) Lint & Epling	< DL	0.074 ± 0.002	1.72 ± 0.44	13.5 ± 0.7	9.1 ± 0.4	0.26 ± 0.05	0.23 ± 0.16	6.97 ± 0.90	0.024 ± 0.003	10.7 ± 1.9
*Ageratum houstonianum* Mill.	0.210 ± 0.005	0.148 ± 0.009	4.34 ± 0.41	72.5 ± 0.2	44.1 ± 1.0	0.41 ± 0.06	0.32 ± 0.17	27.35 ± 0.89	0.079 ± 0.047	34.2 ± 2.0
*Antirrhinum majus* L.	<DL	0.036 ± 0.006	3.55 ± 0.21	33.9 ± 6.2	32.2 ± 0.9	0.34 ± 0.30	0.28 ± 0.07	5.83 ± 1.13	0.048 ± 0.039	22.6 ± 1.6
*Begonia cucullata* var. *cucullata*	<DL	0.251 ± 0.050	7.40 ± 0.58	67.4 ± 1.9	59.6 ± 3.8	1.37 ± 0.13	0.41 ± 0.29	28.91 ± 1.40	0.066 ± 0.024	34.7 ± 3.1
*Cucurbita moschata* Duchesne	<DL	0.143 ± 0.003	18.10 ± 0.21	115.9 ± 5.1	25.7 ± 0.3	1.51 ± 0.17	0.28 ± 0.07	37.89 ± 0.46	0.176 ± 0.001	63.6 ± 0.1
*Cucurbita pepo* L.	<DL	0.079 ± 0.014	19.61 ± 3.32	59.9 ± 6.4	16.3 ± 8.9	0.50 ± 0.09	0.29 ± 0.14	73.84 ± 25.47	0.100 ± 0.085	69.6 ± 4.1
*Dahlia pinnata* Cav.	0.225 ± 0.009	0.082 ± 0.017	7.73 ± 0.48	130.0 ± 8.2	83.9 ± 4.7	0.42 ± 0.10	0.22 ± 0.11	26.78 ± 1.67	0.048 ± 0.016	46.9 ± 5.3
*Dianthus chinensis* L.	0.068 ± 0.006	0.421 ± 0.008	6.17 ± 0.51	54.6 ± 2.3	33.3 ± 2.1	0.38 ± 0.07	0.06 ± 0.07	13.49 ± 1.47	0.040 ± 0.026	54.7 ± 2.3
*Fuchsia regia* (Vand. ex Vell.) Munz	<DL	<DL	5.21 ± 0.18	34.2 ± 2.1	11.3 ± 0.4	0.26 ± 0.17	0.17 ± 0.15	28.50 ± 1.80	0.074 ± 0.058	22.6 ± 0.5
*Hemerocallis fulva* (Linn.) Linn.	<DL	<DL	5.11 ± 0.57	18.8 ± 2.6	13.1 ± 0.6	3.61 ± 0.53	0.25 ± 0.18	5.66 ± 0.69	0.042 ± 0.025	30.3 ± 1.9
*Hibiscus sabdariffa* Linn.	0.079 ± 0.004	<DL	5.88 ± 0.09	14.0 ± 0.7	8.1 ± 0.1	0.82 ± 0.01	0.28 ± 0.03	21.42 ± 0.11	0.045 ± 0.027	25.8 ± 0.2
*Monarda didyma* L.	<DL	<DL	2.61 ± 0.20	10.5 ± 1.2	2.9 ± 0.2	0.37 ± 0.02	0.35 ± 0.35	6.85 ± 0.75	0.041 ± 0.027	9.0 ± 2.0
*Nepeta × faassenii* Bergmans ex Stearn	<DL	0.116 ± 0.008	9.92 ± 0.75	47.5 ± 1.0	39.8 ± 0.5	0.81 ± 0.61	0.28 ± 0.10	42.91 ± 0.62	0.049 ± 0.007	45.1 ± 1.5
*Ocimum x africanum* Lour.	<DL	0.110 ± 0.005	2.22 ± 0.10	50.9 ± 0.9	38.9 ± 0.1	0.22 ± 0.21	0.26 ± 0.01	16.35 ± 0.05	0.050 ± 0.015	40.0 ± 4.9
*Ocimum basilicum* L.	<DL	0.442 ± 0.100	16.62 ± 1.25	63.2 ± 7.9	23.3 ± 2.6	0.39 ± 0.08	0.30 ± 0.15	53.00 ± 4.81	0.065 ± 0.040	58.5 ± 1.8
*Salvia discolor* Kunth	<DL	0.031 ± 0.001	2.35 ± 0.37	12.0 ± 0.3	5.5 ± 1.6	0.34 ± 0.20	0.28 ± 0.06	3.32 ± 0.08	0.046 ± 0.035	10.7 ± 0.2
*Salvia elegans* Vahl	<DL	0.085 ± 0.002	9.62 ± 0.49	20.5 ± 1.1	8.6 ± 0.2	0.58 ± 0.01	0.33 ± 0.14	8.87 ± 0.00	0.050 ± 0.006	19.5 ± 0.1
*Salvia farinacea* Benth.	0.035 ± 0.005	0.145 ± 0.013	5.83 ± 0.30	39.1 ± 0.7	24.5 ± 0.8	0.46 ± 0.10	0.37 ± 0.04	20.39 ± 0.05	0.040 ± 0.032	50.0 ± 0.1
*Salvia greggii* A.Gray	<DL	0.180 ± 0.020	9.14 ± 0.23	31.0 ± 0.3	14.1 ± 0.6	0.36 ± 0.22	0.43 ± 0.04	7.36 ± 0.08	0.040 ± 0.006	28.3 ± 0.4
*Salvia microphylla* Kunth	<DL	0.122 ± 0.004	5.00 ± 0.36	15.5 ± 1.0	23.9 ± 0.9	0.19 ± 0.16	0.19 ± 0.02	4.00 ± 0.06	0.036 ± 0.022	24.2 ± 0.2
*Salvia splendens* Sellow ex Nees	0.105 ± 0.009	0.105 ± 0.004	7.54 ± 0.49	29.7 ± 6.9	17.0 ± 0.7	1.09 ± 0.29	0.89 ± 1.12	13.35 ± 0.67	0.051 ± 0.043	26.8 ± 2.3
*Salvia x jamensis* J. Compton	<DL	0.203 ± 0.014	4.75 ± 0.07	14.4 ± 0.70	8.9 ± 0.2	0.34 ± 0.05	0.23 ± 0.07	3.51 ± 0.07	0.050 ± 0.024	19.8 ± 0.8
*Tagetes erecta* L.	0.076 ± 0.004	0.275 ± 0.020	4.70 ± 0.14	42.3 ± 1.1	86.9 ± 0.7	0.13 ± 0.06	0.28 ± 0.05	10.36 ± 1.03	0.028 ± 0.011	25.6 ± 0.9
*Tagetes lemmonii* A. Gray	0.027 ± 0.001	0.210 ± 0.009	9.57 ± 0.03	54.0 ± 3.1	24.3 ± 0.4	1.37 ± 0.02	0.20 ± 0.01	28.25 ± 0.09	0.059 ± 0.003	38.0 ± 1.3
*Tulbaghia cominsii* Vosa	0.112 ± 0.030	<DL	9.16 ± 0.05	26.5 ± 1.4	24.4 ± 0.6	0.60 ± 0.04	0.17 ± 0.16	1.53 ± 0.07	0.021 ± 0.001	62.3 ± 1.1
*Tulbaghia violacea* Harv.	0.038 ± 0.005	<DL	8.49 ± 0.17	27.7 ± 0.5	16.2 ± 0.2	1.15 ± 0.13	0.29 ± 0.05	2.96 ± 0.20	0.040 ± 0.028	44.0 ± 0.9
*Verbena bonariensis* L.	<DL	<DL	7.62 ± 0.34	19.9 ± 0.3	7.7 ± 0.0	0.38 ± 0.17	0.46 ± 0.10	17.69 ± 0.09	0.025 ± 0.006	23.2 ± 1.0
*Viola odorata L.*	0.212 ± 0.004	0.041 ± 0.004	7.06 ± 0.13	64.0 ± 0.3	51.9 ± 0.0	0.52 ± 0.00	0.49 ± 0.01	6.58 ± 0.13	0.108 ± 0.019	64.1 ± 1.6
**Mean** **± standard deviation**	0.102 ± 0.074	0.160 ± 0.111	7.35 ± 4.40	45.0 ± 32.7	36.7 ± 56.3	0.68 ± 0.68	0.30 ± 0.15	18.62 ± 16.87	0.057 ± 0.033	36.0 ± 17.2
**Median**	0.078	0.133	6.17	34.2	23.9	0.42	0.28	13.49	0.048	34.2
**Range (min-max)**	<DL‒0.225	<DL‒0.442	1.72‒19.61	10.5‒130	2.9‒307.4	0.13‒3.61	0.06‒0.89	1.53‒73.84	0.021‒0.176	9.0‒69.6

<DL = below detection limit (0.020 µg/g for Cd; 0.030 µg/g for Co; 0.07 µg/g for Ni; 0.020 µg/g for V).

**Table 2:** Comparison between the trace element concentrations (average values, as µg/g dry weight) reported in literature and the data of the present study for the same (or similar) floral species. The concentrations expressed on fresh weight basis in the cited literature were converted to dry weight basis (italic).

Species	Ref.	Cd	Co	Cu	Fe	Mn	Ni	Pb	Sr	Zn
			(µg/g Dry Weight)							
*Acmella oleracea* (L.) R.K.Jansen	[110]	ND	ND	17.1	300	107.7	3.6	3.5		62.8
	Present study	0.036	0.214	6.17	122.5	307.4	0.60	0.19	15.94	38.4
*Antirrhinum majus* L.	[9]			*12.86*	*34.76*	*45.48*				*70.56*
	[12]	0.491	0.997	4.10	75.52	9.90	1.282	1.085		13.23
	[10]			*1.97*	*33.8*	*33.8*	*0.18*		*42.5*	*25.2*
	Present study	<DL	0.036	3.55	33.9	32.2	0.34	0.28	5.83	22.6
*Begonia boliviensis*	[9]			*13.66*	*18.66*	*30.63*				*32.39*
*Begonia cucullata* var. *cucullata*	Present study	<DL	0.251	7.40	67.4	59.6	1.37	0.41	28.91	34.7
*Dianthus chinensis* L.	[12]	0.621	1.626	6.36	82.57	18.76	0.805	1.431		31.62
	Present study	0.068	0.421	6.17	54.6	33.3	0.38	0.06	13.49	54.7
*Fuchsia x hybrida*	[9]			*32.14*	*96.67*	*49.64*				*136.31*
*Fuchsia regia* (Vand. ex Vell.) Munz	Present study	<DL	<DL	5.21	34.2	11.3	0.26	0.17	28.50	22.6
*Hemerocallis minor*	[111]			7.8	190	22.0	3.2		42.0	41.3
*Hemerocallis x hybrida*	[12]	0.506	0.956	6.61	37.90	10.01	5.048	1.491		28.26
*Hemerocallis fulva* (Linn.) Linn.	Present study	<DL	<DL	5.11	18.8	13.1	3.61	0.25	5.66	30.3
*Hibiscus sabdariffa* Linn.	[112]			ND	61.4	100				27.1
	[110]	ND	ND	5.6	400	243	3.1	1.8		37.3
	Present study	0.079	<DL	5.88	14.0	8.1	0.82	0.28	21.42	25.8
*Monarda didyma* L.	[12]	0.320	0.941	13.66	165.40	21.24	1.725	0.840		42.76
	Present study	<DL	<DL	2.61	10.5	2.9	0.37	0.35	6.85	9.0
*Salvia elegans* Vahl	[13]	0.0527	0.324	16.3	213	95.3	0.524	0.274		94.3
	Present study	<DL	0.085	9.62	20.5	8.6	0.58	0.33	8.87	19.5
*Tagetes erecta* L.	[9]			*11.60*	*92.77*	*83.62*				*141.38*
	[13]	0.152	0.607	29.4	246	46.8	0.596	0.666		110
	Present study	0.076	0.275	4.70	42.3	86.9	0.13	0.28	10.36	25.6
*Viola x wittrockiana*	[9]			*1.95*	*7.29*	*7.93*				*11.52*
*Viola x wittrockiana*	[10]			*5.60*	*51.7*	*67.7*	*0.86*		*65.8*	*72.6*
*Viola tricolor*	[13]	0.579	0.571	21.1	386	67.4	0.538	0.962		152
*Viola odorata L.*	Present study	0.212	0.041	7.06	64.0	51.9	0.52	0.49	6.58	64.1

**Table 3:** Quality control performed with the two standard reference materials used, NIST1515 (*n* = 7) and BCR-482 (*n* = 4).For each element the measured concentrations (mean ± standard deviation) are compared with the certified values (mean ± half-width of the 95% confidence interval) or with the reference values (mean ± standard deviation) indicated in the certification reports. All concentrations are expressed in μg/g dry weight. Concentration below the detection limit is indicated as < DL. The wavelengths (nm) used in this study and the detection limit (DL) of the method (based on 3 times the standard deviation of blanks, expressed as µg/g dry weight) are also reported.

Element	Wavelength	DL	NIST1515 Certified	NIST1515 Measured	BCR-482 Certified	BCR-482 Measured
	(nm)	(µg/g Dry Weight)				
Cd	226.502	0.020	0.0132 ± 0.0015	< LD	0.56 ± 0.02	0.584 ± 0.017
Co	228.616	0.030	0.09 ^1^	0.08 ± 0.04	0.32 ± 0.03 ^2^	0.287 ± 0.014
Cu	324.754	0.02	5.69 ± 0.13	6.57 ± 0.22	7.03 ± 0.19	7.18 ± 0.28
Fe	259.940	0.5	82.7 ± 2.6	70.5 ± 5.3	804 ± 160 ^2^	692 ± 78
Mn	257.940	0.1	54.1 ± 1.1	58.0 ± 1.9	33.0 ± 0.5 ^2^	29.3 ± 1.1
Ni	231.604	0.07	0.936 ± 0.094	0.906 ± 0.074	2.47 ± 0.07	2.37 ± 0.46
Pb	220.353	0.02	0.47 ± 0.024	0.57 ± 0.16	40.9 ± 1.4	39.7 ± 2.4
Sr	421.552	0.02	25.1 ± 1.1	27.6 ± 1.1	10.35 ± 0.24 ^2^	8.91 ± 0.48
V	311.071	0.020	0.254 ± 0.027	0.224 ± 0.086	3.74 ± 0.61 ^2^	3.43 ± 0.30
Zn	213.856	0.1	12.45 ± 0.43	13.92 ± 0.94	100.6 ± 2.2	94.4 ± 2.4

^1^ Reference value indicated in the certificate of analysis. ^2^ Reference values from [115].

## Supplementary Materials

The following are available online at https://www.mdpi.com/1420-3049/25/12/2891/s1, Table S1: Accepted name, family, synonyms, lifeform, geographical distribution, habitat and common names of the selected plant species. Lifeforms are reported according to *WCSP* (2019). *World Checklist of Selected Plant Families*. Facilitated by the Royal Botanic Gardens, Kew. Available online: http://apps.kew.org/wcsp/ (accessed on 3 April 2020).
