# Supplementary material for: Trace Elements in Edible Flowers from Italy: Further Insights into Health Benefits and Risks to Consumers
Source: Molecules. 2020 Jun 23;25(12):2891. doi: 10.3390/molecules25122891 (PMC7355664; doi:10.3390/molecules25122891)
Supplement: Supplementary file 1 [file molecules-25-02891-s001.pdf]

Table S1. Accepted name, family, synonyms, lifeform, geographical distribution, habitat and common names of the selected plant species. Lifeforms are reported according to *WCSP* (2019). *World Checklist of Selected Plant Families*. Facilitated by the Royal Botanic Gardens, Kew. Available online: http://apps.kew.org/wcsp/ (accessed on 10 June 2020).

**Accepted name and protologue** [22]	**Family** [23]	**Synonyms**	**Lifeform** [24]	**Geographical distribution** [25]	**Habitat**	**Common names**	**References (and other literature therein)**
***Acmella oleracea* (L.) R.K.Jansen (Syst. Bot. Monogr. 8: 65. 1985)**	*Asteraceae* Bercht. & J.Presl	Basionym [22]: *Spilanthes oleracea* L. Syst. Nat., ed. 12. 2: 534 (1767). Heterotypic Synonyms: *Spilanthus oleracea* L.; *Anacyclus pyrethraria* Spreng., Syst. Veg. 3: 497 (1826); *Bidens fervida* Lam., Encycl. 1: 415 (1785); *Bidens fixa* Hook.f., Fl. Brit. India 3(8): 307, sphalm. (1881); *Bidens fusca* Lam., Encycl. 1: 416 (1785); *Bidens oleracea* Cav. ex Steud., Nomencl. Bot. 1: 203 (1840); *Cotula pyretharia* L., Mant. Pl.: 116 (1767); *Isocarpha pyrethraria* (L.) Cass., G.F.Cuvier, Dict. Sci. Nat., ed. 2, 26: 280 (1823); *Pyrethraria dichotoma* Pers. ex Steud., Nomencl. Bot. 2: 420 (1841); *Pyrethrum spilanthus* Medik., Hist. & Commentat. Acad. Elect. Sci. Theod.-Palat. 3: 242 (1775); *Spilanthes acmella* var. *oleracea* (L.) C.B.Clarke ex Hook.f., Fl. Brit. India 3: 307 (1881); *Spilanthes fusca* Lam., Encycl. 1: 416 (1785); *Spilanthes oleracea* L., Syst. Nat., ed. 2, 2: 534 (1767); *Spilanthes oleracea* var. *fusca* (Lam.) DC., Prodr. 5: 624 (1836); *Spilanthes radicans* Schrad. ex DC. Prodr. 5: 624 (1836)	ther.	Native to: Southern South America (Argentina Northwest, Paraguay). Introduced into: Middle Europe (Poland), West Tropical Africa, Asia Tropical, Southern America.	Weedy habitats. Naturalized populations are usually found in wet localities such as lakeside marshes. Alt. 0 - 1500 m	Acmella, Agriao do para, Brede mafane, Brazil cress, Brazilian cress, Eye-ball plant, Eyeball Plant, Jambu, Novocaine plant, Para-cress, Perennial Para Cress, Peek-a-boo, Peek-ABoo Plant, Phak khraat hua van, Salad cree, Spilanthes, Spilanthes cress, Spilanthes plant, Spot Flower, Spot plant, Toothache Plant	[7,23,26-33]
***Agastache aurantiaca* (A.Gray) Lint & Epling (Amer. Midl. Naturalist 33: 225. 1945)**	*Lamiaceae* Martinov	Homotypic Names [24]: *Cedronella aurantiaca* A.Gray, Proc. Amer. Acad. Arts 22: 408 (1886); *Brittonastrum aurantiacum* (A.Gray) Briq. in H.G.A.Engler & K.A.E.Prantl, Nat. Pflanzenfam. 4(3a): 235 (1896).	Hemicr.	Native to: Mexico Northeast	Lowlands (gray or pink-gray igneous out crops in rocky fields, plateaus, and canyon summits in open pine-oak woodlands) Alt.2000-2500 m	Oranssi-iiso, Orangefarbige Duftnessel, Orange hummingbird mint, Jewel of the Sierra Madre, Hyssop, Anise Hyssop	[23,24,32,34]
***Ageratum houstonianum* Mill. (Gard. Dict. ed. 8. Ageratum no. 2. 1768)**	*Asteraceae* Bercht. & J.Presl	*Ageratum conyzoides* Linnaeus var. *Mexicanum* (Sims) de Candolle, Prodr. 5: 108 (1836); *Ageratum conyzoides* var. *houstonianum* (Mill.) T.R.Sahu, Feddes Repert. 93(1-2): 64 (1982); *Ageratum conyzoides* subsp. *houstonianum* (Mill.) M.Sharma Geobios, New Rep. 3(2): 152 (1984); *Ageratum*	Hemicr.	Native to: Mexico Central, Mexico Gulf, Mexico Northeast, Mexico Southeast, Mexico Southwest; Central America (Belize, El Salvador, Guatemala, Honduras). Introduced into: Northern	Pastures, disturbed sites, crops, roadsides, savannas, openings in forests, humid areas and riparian zones. Alt. 0 - 1300 m	Agerate bleu, Blue maudlin, Blue billygoatweed, Bluemink, Blueweed, Celestina azul, Marubio, flossflower, Marrubio, Mexican	[23,32,33,35-42]

Accepted name and protologue [22]	Family [23]	Synonyms	Lifeform [24]	Geographical distribution [25]	Habitat	Common names	References (and other literature therein)
		houstonianum f. *isochroum* (B.L.Rob.) M.F.Johnson, Ann. Missouri Bot. Gard. 58: 25 (1971); *Ageratum mexicanum* Sims, Bot. Mag. 52: t. 2524 (1824); *Ageratum pinetorum* (L.O.Williams) R.M.King & H.Rob.; *Ageratum wendlandii* Vilm.; *Alomia pinetorum* L.O.Williams; *Cacalia mentrasto* Vell.; *Carelia houstoniana* Kuntze		Europe (Great Britain), Middle Europe (Belgium, Germany, Hungary, Poland), Southwestern Europe (France, Portugal,), Southeastern Europe (Bulgaria, Italy, Yugoslavia), Eastern Europe (Belarus, Krym Ukraine); Northern Africa (Egypt), Macaronesia (Azores, Canary Is.) West Tropical Africa (Benin, Mali), West-Central Tropical Africa (Cameroon, Gabon), Northeast Tropical Africa (Chad, Ethiopia, Sudan), South Tropical Africa (Zimbabwe), Southern Africa (Cape Provinces, KwaZulu-Natal Northern Provinces, Swaziland), Western Indian Ocean (Mauritius); Russian Far East (Primorye), Middle Asia (Kazakhstan, Turkmenistan, Uzbekistan), Caucasus (Transcaucasus), China (China North-Central, Hainan, China South-Central, China Southeast); Eastern Asia (Korea, Taiwan); Indian Subcontinent (East Himalaya, India, Nepal, Pakistan, Sri Lanka), Indo-China (Myanmar, Vietnam), Malesia (Jawa, Philippines, Sumatera); Australia (New South Wales, Queensland); Southwestern Pacific (Fiji, New Caledonia), South-Central Pacific (Society Is.), North-Central Pacific (Hawaii); Northeastern USA (Connecticut, Massachusetts, New York), South-Central USA (Texas), Southeastern USA (Alabama, North Carolina, Carolina, Florida, Georgia), Central		paintbrush, Mexikanischer Leberbalsam, Murasakikakkoazami, Ookakkoazami	

Accepted name and protologue [22]	Family [23]	Synonyms	Lifeform [24]	Geographical distribution [25]	Habitat	Common names	References (and other literature therein)
				America (Costa Rica, Nicaragua, Panamá), Caribbean (Cuba, Jamaica, Trinidad-Tobago,), Northern South America (Suriname, Venezuela), Western South America (Bolivia, Colombia, Ecuador, Peru)			
***Antirrhinum majus* L. (Sp. Pl. 2: 617. 1753)**	*Plantaginaceae* Juss.	*Antirrhinum grandiflorum* Stokes, Bot. Mat. Med. 3: 384 (1812); *Antirrhinum hendersonii* Hend., Gard. Chron. 1852: 160 (1852); *Antirrhinum majus* var. *hendersonii* (Hend.) J.Harrison, Fl. Cab. 1852: 217 (1852); *Antirrhinum murale* Salisb., Prodr. Stirp. Chap. Allerton: 98 (1796); *Orontium majus* (L.) Pers., Syn. Pl. 2: 158 (1806)	Herb. phan.	Native to: Northern Africa (Algeria, Libya, Morocco, Tunisia); Asia-Temperate: Western Asia (Cyprus, Israel, Lebanon, Syria, Turkey); Southeastern Europe (Albania, Croatia, Greece (incl. Crete), Italy (incl. Sardinia, Sicily), Malta, Serbia); Southwestern Europe (France (s.), Portugal, Spain (incl. Baleares)).<br>Widely naturalized in temperate regions.<br>Widely cultivated in temperate regions.	Old walls, rocks and dry places, disturbed habitats, common along roadside and railway embankments.<br>Alt. 0 - 800 m	Bocca di leone comune, Boca de dragón, Garden snapdragon, Grand muflier, Gueule de lion, Gueule de loup, Snapdragon, Löwenmaul, Lejongap	[23,32,33,41-44]
Begonia cucullata* var. *cucullata	*Begoniaceae* C.Agardh	Heterotypic Synonyms: *Begonia nervosa* Kunth in F.W.H.von Humboldt, A.J.A.Bonpland & C.S.Kunth, Nov. Gen. Sp. 7: 177 (1825), nom. Nud; *Begonia dispar* Rchb., Mith. Landw.: 54 (1829); *Begonia semperflorens* Link & Otto, Icon. Pl. Rar.: 9 (1829); *Begonia setaria* Graham, Edinburgh New Philos. J. 1829: 180 (1829); *Begonia cucullifolia* Hassk., Hort. Bogor. Descr.: 311 (1858); *Begonia semperflorens f. flavescens C.DC.*, Bull. Herb. Boissier, sér. 2, 3: 405 (1903); *Begonia subcucullata* C.DC., Bull. Herb. Boissier, sér. 2, 3: 404 (1903); *Begonia subcucullata* var. *arenosicola* C.DC., Bull. Herb. Boissier, sér. 2, 3: 404 (1903); *Begonia paludicola* C.DC., Bull. Soc. Bot. Genève, sér. 2, 6: 125 (1914); *Begonia cucullata var. arenosicola* (C.DC.) L.B.Sm. & B.G.Schub., Darwiniana 5: 106 (1941)	Cham. or nanophan.	Native to: South America (Brazil, Argentina, Paraguay, Uruguay, Peru).<br>Known introduced range: Naturalized elsewhere in the tropics	Natural forests, planted forests, ruderal/disturbed, urban areas. Cultivated.<br>Alt. 1-1000 m	Bedding begonia, Begonia, Begonia perpetua, Bégonia semperflorens, Club Begonia, Clubed Begonia, Fibrous Begonia, Paraguayan Begonia, Perpetual Begonia, Sommarbegonia, Wax Semperflorens-Begonie, Semperflorens Begonia, Wax Begonia	[7,23,24,32,33,45-48]
***Cucurbita moschata* Duchesne (Ess. Hist. Nat.**	*Cucurbitaceae* Juss.	*Cucurbita colombiana* (Zhit.) Bukasov, Trudy Prikl. Bot., Suppl. 47: 531 (1930); *Cucurbita hippopera* Ser., Fl. Jard. 2: 531 (1847);	Ther.	Native to: Belize, Guatemala, Mexico Central, Mexico Gulf, Mexico Northeast, Mexico	Warm and moist areas, well-drained fertile grounds. Cultivated in	Abóbora-rasteira, Auyama, Ayote, Bisam-Kürbis, Butternut	[7,23,32,33,41,42, 49-51]

Accepted name and protologue [22]	Family [23]	Synonyms	Lifeform [24]	Geographical distribution [25]	Habitat	Common names	References (and other literature therein)
**Courges: 7. 1786)**		*Cucurbita macrocarpa* Gasp., Rendiconto Reale Accad. Sci. Fis. 6: 447 (1847); *Cucurbita meloniformis* Carrière, Rev. Hort. (Paris) 52: 137 (1880); *Cucurbita moschata* var. *claviformis* Harz, Landw. Samenk. 2: 824 (1885); *Cucurbita moschata* var. *colombiana* Zhit., Trudy Prikl. Bot. 47: 312, 531 (1930); *Cucurbita moschata* var. *depressa* Harz, Landw. Samenk. 2: 824 (1885); *Cucurbita moschata* var. *magna* Harz, Landw. Samenk. 2: 824 (1885); *Cucurbita moschata* var. *meloniformis* (Carrière) L.H.Bailey, Gentes Herbarum 7: 468 (1948); *Gymnopetalum calyculatum* Miq., Fl. Ned. Ind., Eerste Bijv.: 332 (1861); *Pepo eximius* Sageret, Ann. Sci. Nat. (Paris) 8: 311 (1826); *Pepo indicus* Burm., Index Univers. Herb. Amboin. 5: 2 verso (1755); *Pepo macrocarpus* Poit., Jard. Bot. Paris: 246 (1816); *Pepo moschatus* (Duchesne) Britton, Brooklyn Bot. Gard. Mem. 1: 96 (1918)		Southeast, Mexico Southwest Introduced into: Europe: Middle Europe (Austria), Southwestern Europe (Portugal), Southeastern Europe (Albania, Italy); Africa: West-Central Tropical Africa (Cameroon, Zaïre), Northeast Tropical Africa (Sudan), Western Indian Ocean (Chagos Archipelago); Asia Temperate: Middle Asia (Kazakhstan, Uzbekistan, Tadzhikistan, Turkmenistan), Eastern Asia (Korea); Asia Tropical: Indian Subcontinent (Assam, Bangladesh, East Himalaya, India, West Himalaya), Indo-China (Andaman Is., Myanmar, Nicobar Is., Vietnam), Malesia (Sumatera); Northern America: Northeastern USA (Pennsylvania), South-Central USA (Texas), Southeastern USA (Florida, Georgia, Kentucky, Louisiana, Mississippi, North Carolina, South Carolina, Tennessee, Virginia); Southern America: Central America (Costa Rica, El Salvador, Nicaragua, Panamá), Caribbean (Bahamas, Cuba, Dominican Republic, Haiti, Leeward Is., Puerto Rico, Trinidad-Tobago, Venezuelan Antilles, Windward Is.), Northern South America (Venezuela), Western South America (Bolivia, Colombia, Ecuador, Peru), Southern South America (Argentina Northeast)	subtropical and tropical regions worldwide. Alt. 0 - 1500 m	pumpkin, Butternut squash, Calabaza pumpkin, Calabaza moscada, Calabaza pellejo, Cheese pumpkin, Chicamita, Citrouille, Courge musquée, Fak Thong, Golden cushaw, Halva kaddu, Hobag, Kikuza-kabocha, Pumpkin, Lacayote, Loche, Moschuskürbis, Nan gua, Squash, Pâtisson, Red gourd, Spanish gourd, Squash, Squash gourd, Sequaloa, Winter crookneck squash, Zucca torta, Zapallo Pumpkin	
***Cucurbita pepo* L. (Sp. pl. 2:1010. 1753)**	*Cucurbitaceae* Juss.	*Citrullus variegatus* Schrad. ex M.Roem., Fam. Nat. Syn. Monogr. 2: 51 (1846); *Cucumis pepo* (L.) Dumort., Fl. Belg.: 54 (1827); *Cucumis zapallo* Steud., Nomencl. Bot., ed. 2, 1: 452	Ther.	Native to: Mexico Central, Mexico Gulf, Mexico Northeast, Mexico Northwest, Mexico Southeast, Mexico Southwest.	From semi-dry climates and limestone soil to colder climatic conditions and	Bitter bottle gourd, Bush squash, Calabaza Citrouille, Courge pepon, Gemüse-Kürbis,	[7,23,32,33,41,42,51,52]

Accepted name and protologue [22]	Family [23]	Synonyms	Lifeform [24]	Geographical distribution [25]	Habitat	Common names	References (and other literature therein)
		(1840); *Cucurbita aurantia* Willd., Sp. Pl., ed. 4, 4: 607 (1805); *Cucurbita ceratoceras* Haberle ex Mart., Reise Bras. 1: 536 (1823); *Cucurbita clodiensis* Nocca, Fl. Ven. 5: 52; *Cucurbita courgero* Ser., Mém. Soc. Phys. Genève 3(2): t. 1 (1825); *Cucurbita elongata* Bean ex Schrad., Linnaea 12: 407 (1838); *Cucurbita esculenta* Gray, Nat. Arr. Brit. Pl. 2: 552 (1821 publ. 1822); *Cucurbita fastuosa* Salisb., Prodr. Stirp. Chap. Allerton: 157 (1796); *Cucurbita hybrida* Bertol. ex Naudin, Ann. Sci. Nat., Bot., sér. 4, 6: 29 (1856); *Cucurbita lignosa* Mill., Gard. Dict. ed. 8: n.º 5 (1768); *Cucurbita mammeata* Molina, Sag. Stor. Nat. Chili: 133 (1782); *Cucurbita mammosa* J.F.Gmel., Syst. Nat. ed. 13bis: 101 (1791); *Cucurbita oblonga* Link, Handbuch 1: 643 (1829); *Cucurbita pepo* var. *americana* Zhit., Trudy Prikl. Bot. 23(3): 194 (1930); *Cucurbita pepo* var. *bicolor* Harz, Landw. Samenk. 2: 817 (1885); *Cucurbita pepo* var. *elongata* Harz, Landw. Samenk. 2: 816 (1885); *Cucurbita pepo* var. *flogra* Teppner, Phyton (Horn) 40: 35 (2000); *Cucurbita pepo* var. *georgica* Teppner, Phyton (Horn) 40: 35 (2000); *Cucurbita pepo* subsp. *gumala* Teppner, Phyton (Horn) 40: 34 (2000); *Cucurbita pepo* subvar. *nigra* Harz, Landw. Samenk. 2: 815 (1885); *Cucurbita pepo* var. *praecox* Harz, Landw. Samenk. 2: 819 (1885); *Cucurbita pepo* var. *vulgaris* Harz, Landw. Samenk. 2: 818 (1885); *Cucurbita polymorpha* Duchesne, J.B.A.M.de Lamarck, Encycl. 2: 152 (1786); *Cucurbita pomiformis* M.Roem., Fam. Nat. Syn. Monogr. 2: 85 (1846); *Cucurbita pyridaris* Duchesne ex Poir., G.-F.Cuvier, Dict. Sci. Nat., ed. 2, 11: 237 (1819); *Cucurbita pyxidaris* DC., J.B.A.M.de Lamarck & A.P.de Candolle, Fl. Franç., ed. 3, 3: 693 (1805); *Cucurbita succado* Nägeli ex Naudin, Ann. Sci. Nat., Bot., sér. 4, 6: 29 (1856); *Cucurbita succedo* Arn., J. Bot. (Hooker) 3: 278 (1841); *Cucurbita tuberculosa* Schrad., Linnaea 10(Litt.): 70 (1836); *Cucurbita urnigera* Schrad., Linnaea		Introduced into: Europe: Northern Europe (Sweden), Middle Europe (Belgium, Czechoslovakia,), Southwestern Europe (France), Southeastern Europe (Albania, Greece, Italy); Africa: Macaronesia (Cape Verde), West Tropical Africa (Benin, Burkina, Gambia, Nigeria, Sierra Leone), West-Central Tropical Africa (Cameroon, Zaïre), Middle Atlantic Ocean (St.Helena); Asia-Temperate: Middle Asia (Kazakhstan, Uzbekistan, Tadzhikistan, Turkmenistan), Western Asia (Turkey), Arabian Peninsula (Oman), Eastern Asia (Korea), Asia-Tropical: Indian Subcontinent (Assam, Bangladesh, East Himalaya, India, West Himalaya), Indo-China (Vietnam); Northern America: Eastern Canada (Ontario, Québec), North-Central USA (Illinois, Kansas), Northeastern USA (Connecticut, Massachusetts, Michigan New Hampshire, New York, Ohio, Pennsylvania, Vermont), Southwestern USA (California, Nevada, Utah), South-Central USA (New Mexico, Texas), Southeastern USA (Alabama, Arkansas, Kentucky, Louisiana North Carolina, South Carolina, Tennessee, Virginia); Southern America: Central America (Costa Rica, Guatemala), Caribbean (Bahamas, Cayman Is., Cuba, Dominican Republic, Haiti, Puerto Rico, Trinidad-Tobago, Venezuelan Antilles), Northern	sometimes highly eroded soil. Cultivated in temperate, subtropical and tropical regions worldwide. The varieties show good ability of to adapt to different environments. Alt. 0 -2300 m	Ghia kaddu, Güicoy, Ikoy, Marrow, Mensejo, Mucum, Pumpa, Pumpkin, Spaghetti squash, Squash, Summer Squash, Tsol, Xi hu lu, Warron, Winter squash, Zucchino	

Accepted name and protologue [22]	Family [23]	Synonyms	Lifeform [24]	Geographical distribution [25]	Habitat	Common names	References (and other literature therein)
		10(Litt.): 110 (1836); *Cucurbita variegata* Steud., Nomencl. Bot., ed. 2, 1: 452 (1840); *Cucurbita venosa* Descourt., Fl. Méd. Antilles 5: 79 (1827); *Pepo aurantius* (Willd.) Peterm., Pflanzenreich: 438 (1838-1845); *Pepo citrullus* Sageret, Ann. Sci. Nat. (Paris) 8: 311 (1826); *Pepo clypeiformis* Poit., Jard. Bot. Paris: 246 (1816); *Pepo oblongus* Poit., Jard. Bot. Paris: 246 (1816); *Pepo potiron* Sageret, Ann. Sci. Nat. (Paris) 8: 311 (1826); *Pepo vulgaris* Moench, Methodus: 653 (1794)		South America (Venezuela), Western South America (Bolivia), Brazil (Brazil South), Southern South America (Argentina Northeast, Argentina Northwest); Australasia: New Zealand (New Zealand North, New Zealand South); Pacific: Southwestern Pacific (Nauru, New Caledonia, Tokelau-Manihiki, Tuvalu), [53]South-Central Pacific (Cook Is.)			
***Dahlia pinnata* Cav. (Icon. 1: 57.1791)**	*Asteraceae* Bercht. & J.Presl	*Bidens variabilis* Baill., Hist. Pl. 8: 305 (1882); *Coreopsis crassifolia* Sessé & Moc., Fl. Mexic.: 194 (1894), Fl. Mexic.: 194 (1894); *Coreopsis georgina* Cass., Dict. Sci. Nat., ed. 2. 18: 441 (1821); *Dahlia astrantiiflora* G.Don, Hort. Brit. 353 (1839); *Dahlia mexicana* Anon., Vilm. Blumengärtn. ed. 3 1: 489 (1894); *Dahlia nana* Andrews, Bot. Repos. 7: t. 483 (1807); *Dahlia pinnata* var. *nana* Andrews, Bot. Repos. 7: t. 483 (1807); *Dahlia pinnata* var. *variabilis* (Desf.), Voss Vilm. Blumengärtn. ed. 3 1: 489 (1894); *Dahlia purpurea* Poir., J.B.A.M.de Lamarck, Encycl., Suppl. 2: 444 (1812); *Dahlia pusilla* Zucc. ex DC., Prodr. 5: 494 (1836); *Dahlia rosea* Cav., Icon. 3: 33; *Dahlia royleana* Knowles & Westc., 3: 186 (1840); *Dahlia sambucifolia* Salisb., Parad. Lond.: t. 16; *Dahlia sphondyliifolia* Salisb., Trans. Hort. Soc. London 1: 91 (1812); *Dahlia superflua* (DC.) W.T.Aiton, Hortus Kew. 5: 87 (1813); *Dahlia variabilis* Desf., Tabl. École Bot.: 182 (1829); *Georgia variabilis* Spreng., Syst. Veg. 3: 610 (1826); *Georgina astrantiiflora* Sweet, Hort. Brit.: 310 (1830); *Georgina purpurea* Willd., Sp. Pl., ed. 4, 3: 2124 (1803); *Georgina rosea* (Cav.) Willd., Sp. Pl., ed. 4, 3: 2142 (1803); *Georgina superflua* DC., Ann. Mus. Natl. Hist. Nat. 15: 310 (1810); *Georgina superflua* var. *flavescens* DC., Ann. Mus. Natl. Hist. Nat. 15: 310, 311 (1810); *Georgina superflua* var. *lilacina* DC., Ann. Mus. Natl. Hist. Nat. 15: 310 (1810);	Geophyte	Native to: Mexico Central, Mexico Gulf, Mexico Northeast, Mexico Southwest Introduced into: Europe: Northern Europe (Ireland, Great Britain, Norway), Middle Europe (Germany, Hungary, Poland), Southwestern Europe (Baleares, France, Portugal, Spain), Southeastern Europe (Bulgaria, Italy, Romania, Yugoslavia), Eastern Europe (Baltic States, Krym, Ukraine); Africa: West - Central Tropical Africa (Cameroon), Northeast Tropical Africa (Ethiopia); Asia-Temperate: Middle Asia (Kazakhstan, Tadzhikistan, Turkmenistan, Uzbekistan), Caucasus (Transcaucasus); Asia Tropical: Indian Subcontinent (India, East Himalaya, Nepal, Pakistan), Indo-China (Myanmar, Vietnam); Pacific: Southwestern Pacific (Fiji), South-Central Pacific (Cook Is., Tubuai Is.); Northern America: Mexico (Mexico Southeast); Southern America: Central America (Belize, Costa Rica, Guatemala, Honduras, Panamá,	Rich damp oak and pine woods. Alt.1500-4300 m	Dahlia, Dahlia des jardins, Dahlie, Dalria Garden dahlia, Garten-Dahlie, Georgina, Georgine, Havedahlia, Tarhadaalia	[23,32,33,41,53-56]

Accepted name and protologue [22]	Family [23]	Synonyms	Lifeform [24]	Geographical distribution [25]	Habitat	Common names	References (and other literature therein)
		Georgina superflua var. *pallida* DC., Ann. Mus. Natl. Hist. Nat. 15: 310 (1810); *Georgina superflua* var. *purpurea* DC., Ann. Mus. Natl. Hist. Nat. 15: 310, 311 (1810); *Georgina superflua* var. *rubra* DC., Ann. Mus. Natl. Hist. Nat. 15: 310, 311 (1810); *Georgina variabilis* Willd., Hort. Berol. 2(8): t. 93 (1809); *Georgina variabilis* var. *lilacina* (DC.) Willd., Hort. Berol. 2(9): t. 94 (1812); *Georgina variabilis* var. *pallida* (DC.) Willd., Hort. Berol. 2(9): t. 95 (1812); *Georgina variabilis* var. *purpurea* (DC.) Willd., Hort. Berol. 2(9): t. 93 (1812)		Nicaragua), Caribbean (Cuba, Leeward Is., Puerto Rico), Western South America (Colombia, Ecuador)			
***Dianthus chinensis* L. (Sp. Pl.: 411. 1753)**	*Caryophyllaceae* Juss.	Autonym: *Dianthus chinensis* subsp. *chinensis* L. Heterotypic synonyms: *Dianthus amurensis* Jacques, J. Soc. Imp. Centr. Hort. 7: 625 (1861); *Dianthus chinensis* var. *amurensis* (Jacques) Kitag., Neolin. Fl. Manshur.: 266 (1979); *Dianthus chinensis* var. *jingpoensis* G.Y.Zhang & X.Y.Yuan, Bull. Bot. Res., Harbin 18: 10 (1998); *Dianthus chinensis* var. *liaotungensis* Y.C.Chu, Fl. Pl. Herb. Chin. Bor.-Or. 3: 227 (1975); *Dianthus chinensis* var. *morii* (Nakai) Y.C.Chu, Fl. Pl. Herb. Chin. Bor.-Or. 3: 49 (1975); *Dianthus chinensis* var. *serpens* Y.N.Lee, Bull. Korea Pl. Res. 4: 29 (2004); *Dianthus chinensis* var. *shandongensis* J.X.Li, F.Q.Zhou, Bull. Bot. Res., Harbin 21: 511 (2001); *Dianthus chinensis* var. *subulifolius* (Kitag.) Ma, Fl. Intramongolica 2: 191 (1978 publ. 1979); *Dianthus chinensis* var. *versicolor* (Fisch. ex Link) Ma *Dianthus chinensis* subsp. *versicolor* (Fisch. ex Link) Vorosch., A.K.Skvortsov (ed.), Florist. Issl. Razn. Raionakh SSSR: 167 (1985); *Dianthus dentosus* Fisch. ex Rchb., Iconogr. Bot. Pl. Crit. 6: 32 (1828); *Dianthus fischeri* Spreng., Pl. Min. Cogn. Pug. 2: 62 (1815); *Dianthus ibericus* Willd., Enum. Pl. Hort. Berol., Suppl.: 24 (1814); *Dianthus jeniseensis* Less. ex Ledeb., Fl. Ross. 1: 278 (1842); *Dianthus laciniatus* Makino, Bot. Mag. (Tokyo) 17: 60 (1903); *Dianthus morii* Nakai,	Hemicr.	Native to: Europe: Eastern Europe (Krym, Central European Russia, East European Russia, South European Russia, Northwest European Russia, Ukraine); Asia-Temperate: Siberia, Russian Far East (Amur, Khabarovsk, Primorye), Middle Asia (Kazakhstan, Turkmenistan), China (China North-Central, China South-Central, China Southeast, Inner Mongolia, Manchuria, Qinghai, Xinjiang), Mongolia, Eastern Asia (Korea); Asia-Tropical: Indian Subcontinent (East Himalaya), Indo-China (Vietnam). Introduced into: Europe: Middle Europe (Czechoslovakia); Asia Temperate: Russian Far East (Magadan), Middle Asia (Tadzhikistan, Uzbekistan); Asia-Tropical: Indian Subcontinent (Bangladesh). Cultivated across temperate regions of the world.	Sandy forest margins, forest grasslands, scrub on mountain slopes, hillside grasslands, dry hillsides, sandy hill summits, valleys, rocky ravines, meadows, streamsides, mountain stream wetlands, mountain waterfalls, rocks, steppes, steppe sands, fixed dunes, seashores. Widely cultivated in temperate regions, and tropical and subtropical areas.	Annual pink, China pink, Chinenser-Nelke, Clavel, Clavellina, Dianthus, Indian pink, Japanese pink, Japanese Rōmaj, Kaiser-Nelke, Kara-nadeshiko, Oeillet de Chine, Paeraenikkot Pink, Rainbow pink, Shi zhu, Sekichiku, Sommarnejlika, Zaw-hmwa-gale	[32,33,41,57,58]

Accepted name and protologue [22]	Family [23]	Synonyms	Lifeform [24]	Geographical distribution [25]	Habitat	Common names	References (and other literature therein)
		Bot. Mag. (Tokyo) 28: 302 (1914); *Dianthus patens* Willd., Enum. Pl. Hort. Berol., Suppl.: 24 (1814); *Dianthus pineticola* Kleopow, Izv. Kievsk. Bot. Sada 12-13: 161 (1931); *Dianthus pulcher* Salisb., Prodr. Stirp. Chap. Allerton: 303 (1796); *Dianthus ruthenicus* Roem. ex Poir., J.B.A.M.de Lamarck, Encycl., Suppl. 4: 131 (1816); *Dianthus schraderi* Rchb., Iconogr. Bot. Exot. 1: 24 (1824); *Dianthus subulifolius* Kitag., Rep. Exped. Manchoukuo Sect. IV 2: 16 (1935); *Dianthus tataricus* Fisch., Cat. Jard. Gorenki, ed. 2: 59 (1812); *Dianthus umbellatus* DC., Cat. Pl. Horti Monsp.: 104 (1813); *Dianthus versicolor* Fisch. ex Link, Enum. Hort. Berol. Alt. 1: 420 (1821); *Dianthus versicolor* subsp. *ninelli* (Peschkova) Baikov & N.V.Vlassova, Rast. Mir Aziatsk. Rossii 3(23): 21 (2016); *Dianthus versicolor* var. *ninelli* Peschkova, Fl. Centr. Sibiri 1: 334 (1979); *Dianthus versicolor* var. *subulifolius* (Kitag.) Y.C.Chu, Fl. Pl. Herb. Chin. Bor.-Or. 3: 50 (1975); *Dianthus willdenowii* Link, Enum. Hort. Berol. Alt. 1: 420 (1821)					
Fuchsia regia **(Vand. ex Vell.) Munz (Proc. Calif. Acad. Sci., ser. 4, 25: 13. 1943)**	*Onagraceae* Juss.	*Fuchsia integrifolia* Cambess., A.F.C.de Saint-Hilaire, Fl. Bras. Merid. 2: 273 (1830); *Fuchsia pyrifolia* C.Presl, Symb. Bot. 2: 19 (1834); *Quelusia regia* Vand. ex Vell., Fl. Flumin.: 149 (1829); *Fuchsia regia* subsp. *regia*	Nanophan.	Native to: Southern America: Brazil (Brazil Northeast, Brazil Southeast)<br>Introduced into: Asia Tropical: Indian Subcontinent (Sri Lanka)	Cloud forests, ravines and edges of forests.<br>Alt.700 - 2000 m	Brinco de princesa, Bucket Flower, Fuchsia, Ladies' Eardrops	[32,33,41,59-61]
Hemerocallis fulva **(Linn.) Linn. (Sp. Pl., ed. 2. 1: 462. 1762)**	*Asphodelaceae* Juss.	Homotypic Names:<br>Basionym/Replaced Synonym: *Hemerocallis lilioasphodelus* var. *fulva* L., Sp. Pl.: 324 (1753); *Hemerocallis crocea* Lam., Fl. Franç. 3: 267 (1779), nom. superfl.	Hemicr. or rhizome geophyte	Native to: Asia-Temperate: China (China North-Central, China South-Central, China Southeast,); Eastern Asia (Japan, Korea, Nansei-shoto, Taiwan).<br>Introduced into: Europe: Middle Europe (Belgium, Germany, Switzerland), Eastern Europe (Central European Russia, Ukraine), Southeastern Europe (Romania, Yugoslavia); Asia Temperate: Russian Far East (Primorye), Caucasus (North Caucasus), Western Asia (Turkey), Middle Asia	Roadsides, waste places, homesteads, open forests, stream banks.<br>Alt. 300-2500 m	Fulvous daylily, Hémérocalle fauve, Lis d’un jour, Orange daylily, Tawny daylily, Xuan cao	[7,23,32,33,41,42]

Accepted name and protologue [22]	Family [23]	Synonyms	Lifeform [24]	Geographical distribution [25]	Habitat	Common names	References (and other literature therein)
				(Kirgizstan); Asia Tropical: Indian Subcontinent (Pakistan); Australasia: New Zealand (New Zealand North, New Zealand South); Northern America: Southeastern USA, Mexico (Mexico Central, Mexico Northeast, Mexico Southwest, Mexico Southeast); Northern America: Eastern Canada (Québec, Nova Scotia, New Brunswick, Prince Edward I.), Northwestern USA (Colorado, Idaho, Montana, Ontario, Washington), North-Central USA (Illinois, Iowa, Kansas, Minnesota, Missouri, Nebraska, South Dakota, Wisconsin), Northeastern USA (Connecticut, Indiana, Maine, Massachusettes, Michigan, New Hampshire, New Jersey, New York, Ohio, Pennsylvania, Rhode I., Vermont, West Virginia), Southwestern USA (Utah), South-Central USA (Texas); Southern America: Central America (Costa Rica, Honduras, Panamá), Caribbean (Cuba, Dominican Republic, Haiti, Leeward Is., Puerto Rico Jamaica, Windward Is.). Widely cultivated as an ornamental.			
***Hibiscus sabdariffa* Linn. (Sp. Pl. 695. 1753. not 1737, 1747, 1759 and 1763)**	*Malvaceae* Juss.	*Abelmoschus cruentus* (Bertol.) Walp., Repert. Bot. Syst. 1: 310 (1842); *Furcaria sabdariffa* Ulbr., H.G.A.Engler & C.G.O.Drude, Veg. Erde 9(III 2): 402 (1921); *Hibiscus cruentus* Bertol., Fl. Guatimal.: 28 (1840); *Hibiscus digitatus* Cav., Diss. 3: 151 (1787); *Hibiscus digitatus* var. *kerrianus* DC., Prodr. 1: 453 (1824); *Hibiscus fraternus* L. Pl. Surin.: 90 (1775); *Hibiscus gossypifolius* Mill.,	ther.	Native to: Africa: West Tropical Africa (Ghana, Nigeria), West-Central Tropical Africa (Central African Republic, Congo, Gabon, Zaïre), Northeast Tropical Africa (Chad, Sudan). Introduced into: Africa: Northern Africa (Egypt), West Tropical Africa (Benin, Burkina,	Spinifex shrublands, sandy streamlines and disturbed sites. Alt. 0 - 1700 m	Acedera de Guinea, Carurú-de-Guiné, Guinée, Indian-sorrel, Jamaica-sorrel, Malventee, Red-sorrel; Roselle, Sorrel, Osa de Jamaica, Oseille de Rosella, Quiabo-azedo, Quiabo-de-Angola,	[23,32,33,41,62-64]

Accepted name and protologue [22]	Family [23]	Synonyms	Lifeform [24]	Geographical distribution [25]	Habitat	Common names	References (and other literature therein)
		Gard. Dict. ed. 8: n.º10 (1768); *Hibiscus masuianus* De Wild. & T.Durand, Bull. Soc. Roy. Bot. Belgique, Compt. Rend. 38: 20 (1899); *Hibiscus palmatilobus* Baill., Bull. Mens. Soc. Linn. Paris 1: 509 (1885); *Hibiscus sanguineus* Griff., Not. Pl. Asiat. 4: 520 (1854); *Sabdariffa digitata* (Cav.) Kostel., Allg. Med.-Pharm. Fl. 5: 1857 (1836); *Sabdariffa rubra* Kostel., Allg. Med.-Pharm. Fl. 5: 1857 (1836)		Gambia, Guinea, Guinea-Bissau, Mali, Mauritania, Niger, Senegal, Sierra Leone, Togo), West-Central Tropical Africa (Cameroon, Gulf of Guinea Is.), Northeast Tropical Africa (Somalia), East Tropical Africa (Tanzania), South Tropical Africa, Southern Africa (Namibia, Northern Provinces), Western Indian Ocean (Réunion, Mauritius,); Asia Temperate: China (Hainan, China South-Central, China Southeast), Eastern Asia (Taiwan); Asia Tropical: Indian Subcontinent (Bangladesh, East Himalaya, India, Sri Lanka), Indo-China (Andaman Is., Cambodia, Laos Thailand, Vietnam), Papuasia (Solomon Is.); Pacific: Southwestern Pacific (Fiji), Northern America: Mexico (Mexico Central, Mexico Gulf, Mexico Northeast, Mexico Northwest, Mexico Southeast, Mexico Southwest); Southern America: Central America (Belize, Guatemala, El Salvador), Caribbean (Cuba, Cayman Is., Dominican Republic, Haiti, Jamaica, Leeward Is., Puerto Rico, Southwest Caribbean, Trinidad-Tobago, Windward Is.), Northern South America (Venezuela), Western South America (Colombia, Peru), Brazil (Brazil Southeast)		Quiabo-róseo, Quiabo-roxo, Rosela, vinagreira, Serení, Rosellhibiskus	
***Monarda didyma* L. (Sp. Pl.: 22. 1753)**	*Lamiaceae* Martinov	Heterotypic Synonyms: *Monarda purpurea* Lam., Encycl. 4: 256 (1797); *Monarda coccinea* Michx., Fl. Bor.-Amer. 1: 16 (1803); *Monarda didynama* Stokes, Bot. Mat. Med. 1: 44 (1812), orth. var.; *Monarda*	Hemicr.	Native to: Northern America: North-Central USA (Illinois), Northeastern USA (Connecticut, Maine, Michigan Massachusetts, New Hampshire, New Jersey,	Moist, open woods, meadows, seepage areas, stream banks, waste areas, roadside ditches, mountains.	Beebalm, Bergamot, Fragrant balm, Fragrantbalm, Mountain-mint, Oswego-tea, Scharlach-	[23,24,32,33,41,65-67]

Accepted name and protologue [22]	Family [23]	Synonyms	Lifeform [24]	Geographical distribution [25]	Habitat	Common names	References (and other literature therein)
		kalmiana Pursh, Fl. Amer. Sept. 1: 17 (1813); *Monarda oswegoensis* W.P.C.Barton, Fl. Philadelph. Prodr. 1: 34 (1815); *Monarda didyma* var. *angustifolia* Torr., Fl. N. Middle United States 1: 25 (1824); *Monarda purpurascens* Wender., Index Seminum (MB, Marburgensis) 1828: [4] (1828); *Monarda contorta* C.Morren, Belgique Hort. 1: 37 (1851).		New York, Ohio, Pennsylvania, Vermont, West Virginia), Southeastern USA (Alabama, Georgia, Kentucky, Maryland, Mississippi, Tennessee, Virginia), Southeastern U.S.A. (North Carolina), Eastern Canada (Ontario). Introduced into: Europe: Middle Europe (Austria), Eastern Europe (Central European Russia)	Alt.1000-1980 m	Indianernessel, Scharlach-Goldmelisseröd, temynta, Scarlet beebalm	
***Nepeta x faassenii* Bergmans ex Stearn (J. Roy. Hort. Soc. 75: 405. 1950) (unplaced name)**	*Lamiaceae* Martinov	This species is an artificial hybrid. Hybrid formula: *N. nepetella* × *N. racemosa*. Hybrid Parent: *Nepeta racemosa* Lam., Encycl., J. Lamarck & al.] 1(2): 711 (1785); Hybrid Parent: *Nepeta nepetella* L., Syst. Nat., ed. 10. 2: 1096 (1759).	Herb. phan.	Only cultivated.	-	Catmint, Faassen's catnip, Hybrid-Katzenminze, Grijs kattenkruid, Almindelig blåkant, Kantnepeta, šanta zkřížená, šanta Fassenova, šanta modrá, šanta Mussiniho	[23,24,32,33,64]
***Ocimum* × *africanum* Lour. (Fl. Cochinch.: 370. 1790)**	*Lamiaceae* Martinov	Heterotypic Synonyms: *Ocimum* × *pilosum* Willd., Enum. Pl.: 629 (1809); *Ocimum* × *pilosum* Roxb., Asiat. Res. 11: 173 (1810), nom. illeg.; *Ocimum basilicum* var. *pilosum* (Willd.) Benth. in N.Wallich, Pl. Asiat. Rar. 2: 13 (1830); *Ocimum basilicum* var. *anisatum* Benth., Labiat. Gen. Spec.: 4 (1832); *Ocimum* × *citriodorum* Vis., Index Seminum (PAD, Patavium) 1840: 9 (1840); *Ocimum* × *graveolens* A.Br., Flora 24: 265 (1841); *Ocimum* × *petitianum* A.Rich., Tent. Fl. Abyss. 2: 176 (1850); *Ocimum americanum* var. *pilosum* (Willd.) A.J.Paton, Kew Bull. 47: 426 (1992).	Ther. or cham.	Native to: Africa: West-Central Tropical Africa (Cameroon, Zaïre), Northeast Tropical Africa (Ethiopia), East Tropical Africa, South Tropical Africa, Southern Africa (Botswana, Northern Provinces), Western Indian Ocean (Madagascar); Asia Temperate: China (China South-Central, China Southeast), Eastern Asia (Taiwan); Asia-Tropical: Indian Subcontinent (India, Sri Lanka), Indo-China (Cambodia, Laos, Myanmar, Thailand, Vietnam), Malesia (Malaya). Introduced into: Morther America: Mexico (Mexico Southeast); Southern America: Central America (Guatemala), Caribbean (Netherlands	Disturbed ground, damp areas Alt. 0 – 1800 (-2250) m	Citronbasilika, Hoary basil, Lemon basil	[23,24,32,33,68,69]

Accepted name and protologue [22]	Family [23]	Synonyms	Lifeform [24]	Geographical distribution [25]	Habitat	Common names	References (and other literature therein)
				Antilles), Brazil (Brazil Northeast)			
***Ocimum basilicum* L. (Sp. Pl. 2: 597. 1753)**	*Lamiaceae* Martinov	Homotypic Name: *Ocimum odorum* Salisb., Prodr. Stirp. Chap. Allerton: 87 (1796), nom. superfl.<br>Heterotypic Synonyms: *Ocimum album* L., Mant. Pl. 1: 85 (1767); *Ocimum anisatum* Benth., Labiat. Gen. Spec.: 4 (1832); *Ocimum basilicum* var. *album* (L.) Benth. in N.Wallich, Pl. Asiat. Rar. 2: 13 (1830); *Ocimum basilicum* var. *densiflorum* Benth., Labiat. Gen. Spec.: 5 (1832); *Ocimum basilicum* var. *difforme* Benth. in N.Wallich, Pl. Asiat. Rar. 2: 13 (1830); *Ocimum basilicum* var. *glabratum* Benth. in N.Wallich, Pl. Asiat. Rar. 2: 13 (1830); *Ocimum basilicum* var. *majus* Benth. in N.Wallich, Pl. Asiat. Rar. 2: 13 (1830); *Ocimum basilicum* var. *purpurascens* Benth. in N.Wallich, Pl. Asiat. Rar. 2: 13 (1830); *Ocimum basilicum* var. *thyrsiflorum* (L.) Benth., Labiat. Gen. Spec.: 5 (1832); *Ocimum basilicum* var. *bullatum* (Lam.) Alef., Landw. Fl.: 114 (1866); *Ocimum basilicum* var. *pelvifolium* Alef., Landw. Fl.: 114 (1866); *Ocimum basilicum* var. *violaceum* Alef., Landw. Fl.: 114 (1866); *Ocimum basilicum* var. *violocrispum* Alef., Landw. Fl.: 114 (1866; *Ocimum basilicum* var. *viridicrispum* Alef., Landw. Fl.: 114 (1866); *Ocimum basilicum* var. *vulgare* Alef., Landw. Fl.: 114 (1866); *Ocimum barrelieri* Roth, Nov. Pl. Sp.: 278 (1821); *Ocimum bullatum* Lam., Encycl. 1: 384 (1785); *Ocimum caryophyllatum* Roxb., Fl. Ind. ed. 1832, 3: 16 (1832); *Ocimum chevalieri* Briq., Mém. Soc. Bot. France 8: 279 (1917); *Ocimum ciliare* B.Heyne ex Hook.f., Fl. Brit. India 4: 608 (1885); *Ocimum ciliatum* Hornem., Hort. Bot. Hafn.: 565 (1815); *Ocimum citrodorum* Blanco, Fl. Filip., ed. 2: 591 (1845); *Ocimum cochleatum* Desf., Tabl. École Bot.: 220 (1804); *Ocimum dentatum* Moench, Methodus: 413 (1794); *Ocimum hispidum* Lam., Encycl. 1: 384 (1785); *Ocimum integerrimum* Willd., Sp. Pl.,	Ther. or cham.	Native to: Asia Temperate: China (China South-Central, China Southeast), Eastern Asia (Taiwan); Asia Tropical: Indian Subcontinent (Assam, Bangladesh East Himalaya, India, Nepal, Sri Lanka, West Himalaya), Indo-China (Andaman Is., Laos, Cambodia, Myanmar, Nicobar Is. Thailand, Vietnam), Malesia (Borneo, Jawa, Lesser Sunda Is., Malaya, Maluku, Philippines, Sulawesi Sumatera), Papuasia (New Guinea)<br>Introduced into: Europe: Eastern (South European Russia, Ukraine), Europe (Africa: Macaronesia (Canary Is., Cape Verde), West Tropical Africa (Benin, Burkina, Ghana, Guinea, Guinea-Bissau, Ivory Coast, Liberia, Mali Niger, Nigeria, Senegal, Sierra Leone), West-Central Tropical Africa (Burundi, Cameroon, Central African Republic, Congo, Gambia, Gabon, Gulf of Guinea Is., Zaïre), Northeast Tropical Africa (Eritrea, Ethiopia, Chad, Sudan), East Tropical Africa, South Tropical Africa, Southern Africa (Cape Provinces),Western Indian Ocean (Comoros, Madagascar); Asia-Temperate: Russian Far East (Primorye), Middle Asia (Kazakhstan), Western Asia (East Aegean Is.), China (Xinjiang), Mongolia; Asia-Tropical: Indian Subcontinent (Laccadive Is.,	Cultivated, not frost-hardy. Cultivated and disturbed ground, ground prone to flooding, grassland Alt. 0 - 1100 m	Basil, Sweet basil	[23,24,32,33,41,42, 68,70]

Accepted name and protologue [22]	Family [23]	Synonyms	Lifeform [24]	Geographical distribution [25]	Habitat	Common names	References (and other literature therein)
		ed. 4, 3: 162 (1800); *Ocimum lanceolatum* Schumach. & Thonn., C.F.Schumacher, Beskr. Guin. Pl.: 268 (1827); *Ocimum laxum* Vahl ex Benth., Labiat. Gen. Spec.: 5 (1832); *Ocimum majus* Garsault, Fig. Pl. Méd.: t. 418a (1764); *Ocimum medium* Mill., Gard. Dict. ed. 8: n.º 3 (1768); *Ocimum minus* Garsault, Fig. Pl. Méd.: t. 418b (1764); *Ocimum nigrum* Thouars ex Benth., Labiat. Gen. Spec.: 5 (1832); *Ocimum odorum* Salisb., Prodr. Stirp. Chap. Allerton: 87 (1796); *Ocimum scabrum* Wight ex Hook.f., Fl. Brit. India 4: 608 (1885); *Ocimum simile* N.E.Br., W.H.Harvey & auct. suc. (eds.), Fl. Cap. 5(1): 234 (1910); *Ocimum thyrsiflorum* L., Mant. Pl. 1: 84 (1767); *Ocimum urticifolium* Benth., Labiat. Gen. Spec.: 5 (1832); *Plectranthus barrelieri* (Roth) Spreng., Syst. Veg. 2: 691 (1825)		Maldives), Papuasia (Bismarck Archipelago); Pacific: Southwestern Pacific (Fiji, Gilbert Is., Samoa, Tokelau-Manihiki, New Caledonia, Niue Tonga, Wallis-Futuna Is.), South-Central Pacific (Cook Is., Line Is., Society Is.), North-Central Pacific (Hawaii); Northern America: North-Central USA (Illinois), Northeastern USA (New York) Mexico (Mexico Central, Mexico Gulf, Mexico Southeast, Mexico Southwest), Southern America: Central America (El Salvador, Guatemala, Honduras, Nicaragua, Panamá), Caribbean (Bahamas, Cayman Is., Cuba, Dominican Republic, Haiti, Jamaica, Leeward Is., Puerto Rico, Trinidad-Tobago, Venezuelan Antilles, Windward Is.), Northern South America (Venezuela), Western South America (Bolivia, Colombia, Ecuador), Brazil (Brazil Southeast).			
***Salvia discolor* Kunth (in F.W.H.von Humboldt, A.J.A.Bonpland & C.S.Kunth, Nov. Gen. Sp. 2: 294 (1818).)**	*Lamiaceae* Martinov	Heterotypic Synonyms: *Salvia bonplandiana* F.Dietr., Nachtr. Vollst. Lex. Gärtn. 7: 403 (1821); *Salvia mexicana* Hemsl., Gard. Chron., n.s., 19: 341 (1883), nom. illeg.; *Salvia nigricans* Hemsl., Gard. Chron., n.s., 19: 341 (1883).	Cham.	Native to: Southern America: Western South America (Peru)	Disturbed areas, rocky slopes, shrublands Alt.1000-2500 m	Andean sage	[23,24,32,33,71-73]
***Salvia elegans* Vahl (Enum. Pl. Obs. 1: 238. 1804)**	*Lamiaceae* Martinov	Heterotypic Synonyms: *Salvia incarnata* Cav., Anales Hist. Nat. 2: 112 (1800), nom. illeg.; *Salvia microculis* Poir. in J.B.A.M.de Lamarck, Encycl. 6: 614 (1805); *Salvia punicea* M.Martens & Galeotti, Bull. Acad. Roy. Sci. Bruxelles 11(2): 65 (1844); *Salvia microcalyx* Scheele, Linnaea 22: 589 (1849); *Salvia*	Cham.	Native to: Northern America: Mexico (Mexico Central, Mexico Gulf, Mexico Northeast, Mexico Northwest, Mexico Southwest)	Pine forests Alt.2800-2900 m	Ananas-Salbei, Ananassalbei, Pineapple sage	[23,24,32,33,41,71,72,74-78]

**Accepted name and protologue** [22]	**Family** [23]	**Synonyms**	**Lifeform** [24]	**Geographical distribution** [25]	**Habitat**	**Common names**	**References (and other literature therein)**
		camertonii Regel, Bot. Zeitung (Berlin) 11: 334 (1853); *Salvia rutilans* Carrière, Rev. Hort. (Paris) 45: 251 (1873); *Salvia longiflora* Sessé & Moc., Pl. Nov. Hisp.: 8 (1887); *Salvia elegans* var. *sonorensis* Fernald, Proc. Amer. Acad. Arts 35: 550 (1900).					
***Salvia farinacea* Benth. (Labiat. Gen. Spec.: 274. 1833)**	*Lamiaceae* Martinov	Heterotypic Synonyms: *Salvia virgata* Ortega, Nov. Rar. Pl. Descr. Dec.: 3 (1797), nom. illeg.; *Salvia trichostyle* Bisch., Index Seminum (HEID, Heidelbergensi) 1847: 4 (1847); *Salvia amabilis* Kunth & C.D.Bouché, Index Seminum (B, Berolinensis) 1848: 13 (1848); *Salvia caesia* Scheele, Linnaea 22: 588 (1849); *Salvia linearis* Sessé & Moc., Fl. Mexic.: 6 (1892), nom. illeg.; *Salvia farinacea* var. *heteranthera* Brandegee, Univ. Calif. Publ. Bot. 4: 187 (1911); *Salvia earlei* Wooton & Standl., Contr. U.S. Natl. Herb. 16: 169 (1913); *Salvia farinacea* var. *latifolia* Shinners, Field & Lab. 21: 92 (1953).	Hemicr. or cham.	Native to: Northern America: South-Central USA, Mexico (Mexico Northeast) Introduced into: Asia-Tropical: Indian Subcontinent (India); Northern America: North-Central USA (Oklahoma Northeastern USA (Connecticut, Ohio), Southeastern USA (Florida, Louisiana); Southern America: Central America (El Salvador, Guatemala), Caribbean (Trinidad-Tobago), Northern South America (Venezuela).	Shrubby areas, oak-pine woodlands and grasslands in mostly calcareous soils. Cultivated and sometimes occurring as a garden throughout Alt.1400-2100 m	Blue sage, Daggsalvia, Härmesalvia, Mealy sage, Mealy-cup sage, Mehliger Salbei, Salvia doméstica, Salvia larga, Salvia mayor, Salva-das-boticas, Salva-mansa, Salva-ordinaria, Salva-trilobada, Sauge farineuse, Texas-Salvie	[23,24,32,33,71,72, 74,76,77,79-82]
***Salvia greggii* A.Gray (Proc. Amer. Acad. Arts 8: 369. 1870)**	*Lamiaceae* Martinov	*Salvia microphylla*, Torr. Bot. Mex. Bound. 131, not HBK.	Cham.	Native to: Northern America: South-Central USA (Texas), Mexico (Mexico Northeast)	Shady hillsides and riverbanks. In Pine and Quercus forests, limestone areas. Alt.1500-2600 m	Autumn sage, Red Chihuahuan sage, Texas red sage.	[32,33,71,72,75,77, 82]
***Salvia microphylla* Kunth (Nov. Gen. Sp. 2: 294. 1818)**	*Lamiaceae* Martinov	*Lasemia coccinea* Raf., 1837, Fl. Tellur. 3: 91. 1837, *Salvia grahamii* Benth. In Bot. Reg., tab. 1370 (1830).	Cham. or nanophan.	Native to: Northern America: Southwestern USA (Arizona), Mexico (Mexico Central, Mexico Gulf, Mexico Northeast, Mexico Northwest, Mexico Southeast, Mexico Southwest); Southern America: Central America (Guatemala). Introduced into: Europe: Southwestern Europe (Corse, France, Portugal, Spain), Southeastern Europe (Greece); Africa: Northern Africa (Morocco, Tunisia); Australasia: New Zealand (New Zealand North); Northern America: South-Central USA (Texas); Southern America: Southern	In mountains, woodland and shaded places. Cultivated as an ornamental and sometimes naturalized in ribazos, near inhabited areas, constructed, industrial and other artificial habitats (50-770 m). Alt.2000-3000 m	Autumn Sage, Baby sage, Blackcurrant Sage, Cherry Sage, Delta Sage, Erva-dos-rapazinhos, Graham's sage, Little Leaf Sage, Sauge à petites feuilles	[23,24,32,41,42,71, 72,75,77,80-85]

Accepted name and protologue [22]	Family [23]	Synonyms	Lifeform [24]	Geographical distribution [25]	Habitat	Common names	References (and other literature therein)
				South America (Argentina Northeast, Argentina Northwest, Uruguay)			
***Salvia splendens* Sellow ex Nees (Flora 4: 300. 1821)**	*Lamiaceae* Martinov	Homotypic Names: *Fenixanthes splendens* (Sellow ex Nees) Raf., Autik. Bot.: 122 (1840); *Horminum splendens* (Sellow ex Nees) M.Gómez, Fl. Habanera: 393 (1897); *Jungia splendens* (Sellow ex Nees) Soják, Cas. Nár. Mus., Odd. Prír. 152: 21 (1983). Heterotypic Synonyms: *Salvia brasiliensis* Spreng., Syst. Veg. 1: 56 (1824); *Salvia colorans* Benth., Labiat. Gen. Spec.: 287 (1833), not validly publ.; *Salvia issanchou* Anon., Rev. Hort. (Paris) 62: 229 (1890).	Cham.	Native to: Southern America: Brazil (Brazil Southeast). Introduced into: Europe: Middle Europe (Czechoslovakia), Eastern Europe (Ukraine); Africa: West Central tropical Africa (Gulf of Guinea Is.); Asia-Temperate: Eastern Asia (Korea); Asia Tropical: Indian Subcontinent (India, Bangladesh); Pacific: Southwestern Pacific (Fiji); Northern America: Southeastern USA (Florida), Mexico (Mexico Southeast); Southern America: Central America (Costa Rica, El Salvador, Guatemala, Honduras, Nicaragua), Caribbean (Cayman Is., Cuba, Jamaica, Puerto Rico, Trinidad-Tobago, Venezuelan Antilles), Northern South America (Venezuela), Western South America (Colombia, Peru), Brazil (Brazil South), Southern South America (Argentina Northeast, Argentina Northwest)	Shady woods, up to the rim of shrubs, shady hill-countries. Widely cultivated as an ornamental. Alt.1000-3100 m	Scarlet sage, Tropical sage	[23,24,32,33,42,71, 72,76,80,86]
***Salvia x jamensis* J. Compton (Plantsman 15: 204. 1994)**	*Lamiaceae* Martinov	This species is a hybrid. Hybrid formula: *S. greggii* × *microphylla*. Hybrid Parent: *Salvia microphylla* Sessé & Moc., Fl. Mexic. 9 (La Naturaleza, Ser. 2, 2: App. 2); ed. 2, 9 (1893). Hybrid Parent: *Salvia greggii* A.Gray, Proc. Amer. Acad. Arts viii. (1870) 369.	Nanophan.	Native to: Northern America: Mexico (Mexico Northeast)	Among bushes and rocks in Mexican mountains, in large thickets in shrubby undergrowth. Alt.2000-3000 m	Jame Sage	[23,24,32,33,85]
***Tagetes erecta* L. (Sp. Pl.: 887. 1753)**	*Asteraceae* Bercht. & J.Presl	*Tagetes corymbosa* Sweet, Brit. Fl. Gard. I: 151 (1829); *Tagetes elongata* Willd., Sp. Pl., ed. 4, 3: 2127 (1803); *Tagetes heterocarpha* Rydb., N. Amer. Fl. 34(2): 155 (1915); *Tagetes major* Gaertn., Fruct. Sem. Pl. 2: 437 (1791); *Tagetes patula* L., Sp. Pl.: 887 (1753); *Tagetes*	Ther.	Native to: Northern America: Mexico (Mexico Central, Mexico Gulf, Mexico Northeast, Mexico Northwest, Mexico Southeast, Mexico Southwest); Southern America: Central America	Lake shore or by streams, Ruderal sites. Alt. 0 - 3000 m	African marigold, Amapola amarilla, Ausgebreitete, Barchatcy otklonennye, Clavelina, Clavelitos de muerto, Copetes,	[7,23,32,33,41,42]

Accepted name and protologue [22]	Family [23]	Synonyms	Lifeform [24]	Geographical distribution [25]	Habitat	Common names	References (and other literature therein)
		remotiflora Kunze, Linnaea 20: 23 (1847).		(Guatemala) Introduced into: Europe: Northern Europe (Ireland, Great Britain, Norway), Middle Europe (Austria, Belgium, Czechoslovakia, Germany, Hungary, Poland), Southwestern Europe (Baleares, France, Portugal, Spain), Southeastern Europe (Bulgaria, Greece, Italy, Romania, Sicilia, Yugoslavia), Eastern Europe (Baltic States, Belarus, Krym, Ukraine); Africa: Northern Africa (Morocco), Macaronesia (Canary Is., Madeira), West Tropical Africa (Benin), West-Central Tropical Africa (Cameroon, Gulf of Guinea Is.,), Northeast Tropical Africa (Chad, Djibouti Ethiopia), South Tropical Africa (Angola, Zambia, Zimbabwe), Northern Provinces, Western Indian Ocean (Mauritius, Réunion, Rodrigues); Asia Temperate: Russian Far East (Primorye), Middle Asia (Kazakhstan, Turkmenistan, Tadzhikistan, Uzbekistan), Caucasus (Transcaucasus), Western Asia (East Aegean Is.), Eastern Asia (Korea, Taiwan); Asia Tropical: Indian Subcontinent (Bangladesh, East Himalaya, India, Laccadive Is., Nepal, Pakistan, Sri Lanka), Indo-China (Andaman Is., Laos, Myanmar, Nicobar Is., Thailand, Vietnam), Malesia (Jawa, Philippines); Pacific: Southwestern Pacific (Fiji, Niue), South-Central Pacific (Cook Is.), Northwestern Pacific (Caroline Is., Marianas, Marshall Is.);		Copetillo, Flor de muerto, French marigold, Koō-sō, Marigold, Pot Marigold, Mansugug, Oeillet d'inde, Osa de muerto, Ruda del pasto, Sammetstagetes, Studentenblume, Studentenblum, ruda, Stinking Suzy, Susi-Susi	

Accepted name and protologue [22]	Family [23]	Synonyms	Lifeform [24]	Geographical distribution [25]	Habitat	Common names	References (and other literature therein)
				Northern America: Northwestern U.S.A. (Wyoming), North-Central USA (Illinois, Kansas, Oklahoma, Missouri, Wisconsin), Northeastern USA (Connecticut, New York, Massachusetts, Ohio Pennsylvania, Vermont), Southwestern USA (California, Utah), Southeastern USA (Alabama, Arkansas, Florida, Kentucky, Louisiana, Maryland, North Carolina, South Carolina, Virginia); Southern America: Central America (Belize, Costa Rica, El Salvador, Honduras Nicaragua, Panamá), Caribbean (Cayman Is., Cuba, Dominican Republic, Haiti, Jamaica, Leeward Is., Puerto Rico, Trinidad-Tobago, Windward Is.), Northern South America (Guyana, Venezuela, Suriname), Western South America.			
***Tagetes lemmonii* A. Gray (Proc. Amer. Acad. Arts 19:40. 1883)**	*Asteraceae* Bercht. & J.Presl	-	Nanophan.	Native to Northern America: Southwestern USA (Arizona), Mexico (Mexico Northeast, Mexico Northwest)	Moist sites, grasslands, scrublands, woodlands, cliffs, streamsides. Alt.1400–2500 m	Lemmon's marigold	[32,33,35,87]
***Tulbaghia cominsii* Vosa (J. S. African Bot. 45: 128. 1979)**	*Amaryllidaceae* J.St.-Hil.	-	Bulb geophyte	Native to: Africa: Sothern Africa (Cape Provinces)	Kommittee soil on rocky ground.	Wild garlic	[23,24,32,33,87-89]
***Tulbaghia violacea* Harv. (Bot. Mag. 64: t. 3555. 1837)**	*Amaryllidaceae* J.St.-Hil.	Homotypic Names: *Omentaria violacea* (Harv.) Kuntze, Revis. Gen. Pl. 2: 713 (1891). *Tulbaghia simmleri* sensu R.B.Burb. in Notes Roy. Bot. Gard. Edin. 36: 98, fig. 3 pro parte (1978), non Beauverd (1908); *Tulbaghia cepacea* L. f., Suppl. Pl. 194 (1781) excl. syn.; Baker in J.L.S. 11: 372 (1871); & in Fl. Cap. 6: 407 (1897); Vosa in Ann. Bot. (Roma) 34: 113, fig. 22, t. 20/1–4 (1975) pro parte, non var. nov., nom. illeg . Type.: South Africa, Cape	Bulb geophyte	Native to: Africa: Sothern Africa (Cape Provinces, KwaZulu-Natal). Introduced into: Africa: East Tropical Africa (Tanzania); Northern America: Mexico (Mexico Central)	Grassland areas. Alt. 0 - 2000 m	Isihaqa, Incinsini, Icinzini (vernacular name), Pink agapanthus, Society garlic Utswelane, Wildeknoffel, Wildeknoflok, Wild garlic	[7,32,33,41,42,89-91]

**Accepted name and protologue** [22]	**Family** [23]	**Synonyms**	**Lifeform** [24]	**Geographical distribution** [25]	**Habitat**	**Common names**	**References (and other literature therein)**
		(LINN. 411.4, holo.)					
***Verbena bonariensis* L. (Sp. Pl.: 20. 1753)**	*Verbenaceae* J.St.-Hil.	Homotypic Names: *Verbena elongata* Salisb., Prodr. Stirp. Chap. Allerton: 71 (1796), nom. superfl.; *Verbena trichotoma* Moench, Suppl. Meth.: 131 (1802), nom. illeg. Heterotypic Synonyms: *Verbena bonariensis* var. longibracteata Kuntze, Revis. Gen. Pl. 3(2): 255 (1898); *Verbena bonariensis* f. *robustior* Chodat, Bull. Herb. Boissier, sér. 2, 2: 817 (1902); *Verbena bonariensis* var. *conglomerata* Briq., Ark. Bot. 2(10): 11 (1904); *Verbena bonariensis* var. *hispida* Rendle, J. Bot. 42: 370 (1904); *Verbena inamoena* Briq., Bull. Herb. Boissier, sér. 2, 4: 1058 (1904); *Verbena intercedens* Briq., Bull. Herb. Boissier, sér. 2, 4: 1057 (1904); *Verbena bonariensis* f. *albiflora* Moldenke, Phytologia 3: 117 (1949); *Verbena bonariensis* var. *hispida* Moldenke, Phytologia 33: 374 (1976), nom. illeg.	Hemicr.	Native to: Southern America: Western South America (Bolivia, Ecuador, Peru), Brazil (Brazil South, Brazil Southeast), Southern South America (Argentina Northeast, Argentina Northwest, Argentina South, Chile Central, Chile North, Chile South, Paraguay, Uruguay). Introduced into: Europe: Northern Europe (Great Britain, Sweden), Middle Europe (Austria, Czechoslovakia, Germany), Southwestern Europe (Portugal, Spain), Southeastern Europe (Italy); Africa: Macaronesia (Azores, Canary Is., Madeira), West-Central Tropical Africa (Zaïre), East Tropical Africa (Kenya, Tanzania), South Tropical Africa (Angola, Mozambique, Zambia, Zimbabwe), Southern Africa (Cape Provinces, Free State, KwaZulu-Natal, Lesotho, Northern Provinces, Swaziland), Middle Atlantic Ocean (St.Helena), Western Indian Ocean (Madagascar, Mauritius, Réunion); Asia Temperate: Eastern Asia (Korea); Asia Tropical: Indian Subcontinent (Assam, India, West Himalaya), Malesia (Borneo, Malaya); Australasia: Australia (Norfolk Is., New South Wales, Northern Territory, Queensland, South Australia, Victoria, Western Australia), New Zealand (New Zealand North, New Zealand South); Pacific: Southwestern Pacific (Fiji, New Caledonia,	In damp soil in grassland, in headwaters of streams and on river banks, in old cultivation and on roadsides. Alt. 65 – 1500 m	Argentinian vervain, Brazilian vervain, Clusterflower vervain, Clustertop vervain, Purpletop vervain, Pretty verbena, Tall verbena, Yerba de fierro	[23,24,32,33,42]

Accepted name and protologue [22]	Family [23]	Synonyms	Lifeform [24]	Geographical distribution [25]	Habitat	Common names	References (and other literature therein)
				Niue), South-Central Pacific (Easter Is.); Northern America: North-Central USA (Illinois, Missouri), Northeastern USA (Massachusetts, New York), Southeastern USA (Alabama, Arkansas, Louisiana, Georgia, Mississippi, North Carolina, South Carolina, Tennessee); Southern America: Caribbean (Bahamas, Bermuda, Jamaica, Puerto Rico); Northern America: Northwestern USA (Oregon), Southwestern USA (California)			
***Viola odorata* L. (Sp. Pl. 2: 934. 1753)**	*Violaceae* Batsch	*Viola austriaca* A.Kern. & Jos.Kern., Ber. Naturwiss.-Med. Vereins Innsbruck 3: 71 (1872); *Viola consimilis* Jord., Mém. Acad. Sci. Lyon, Sect. Sci. 1: 18 (1852); *Viola cyclophylla* Gand., Fl. Lyon.: 53 (1875); *Viola dumetorum* Jord., Mém. Acad. Sci. Lyon, Sect. Sci. 1: 16 (1852); *Viola favratii* Hausskn. ex Favrat, Bull. Murith. Soc. Valais. Sci. Nat. 13-15: 61 (1887); *Viola floribunda* Jord., Mém. Acad. Sci. Lyon, Sect. Sci. 1: 19 (1852); *Viola hortensis* Schur, Enum. Pl. Transsilv.: 82 (1866); *Viola incompta* Jord., Observ. Pl. Nouv. 7: 11 (1849); *Viola jucunda* Jord., Mém. Acad. Sci. Lyon, Sect. Sci. 1: 20 (1852); *Viola maderensis* Lowe, Trans. Cambridge Philos. Soc. 4: 36 (1831); *Viola martia* Hegetschw., Fl. Schweiz: 241 (1839); *Viola odorata* var. *albiflora* Pancic, Verh. Zool.-Bot. Vereins Wien 6: 505 (1856); *Viola odorata* var. *incompta* (Jord.) Espeut, Monde Pl. 464: 15 (1999); *Viola odorata* subsp. *maderensis* (Lowe) G.Kunkel, Cuad. Bot. Canaria 22: 28 (1974); *Viola officinalis* Crantz, Inst. Rei Herb. 2: 431 (1766); *Viola pirottae* Chiov., Bull. Soc. Bot. Ital. 1893: 287 (1893); *Viola plantaginea* Webb ex Christ, Bot. Jahrb. Syst. 9: 96 (1887); *Viola propinqua* Jord., Mém. Acad. Sci. Lyon, Sect. Sci. 1: 18 (1852); *Viola pseudosuavis* Schur, Verh. Naturf. Vereins Brünn 15(2): 103 (1877); *Viola reptans* C.Presl	Hemicr.	Native to: Europe: Northern Europe (Denmark, Ireland, Great Britain, Norway, Sweden) Middle Europe, Southwestern Europe (Corse, France, Portugal, Sardegna, Spain), Southeastern Europe, Eastern Europe (Belarus, Baltic States, Krym, Central European Russia, East European Russia, Northwest European Russia, South European Russia, Ukraine); Africa: Northern Africa (Algeria, Morocco, Tunisia), Macaronesia (Azores, Canary Is., Madeira); Asia-Temperate: Middle Asia (Kazakhstan), Caucasus (Transcaucasus), Western Asia (Cyprus, East Aegean Is., Iran, Iraq, Lebanon-Syria, Palestine, Turkey).<br>Introduced into: Asia-Temperate: Middle Asia (Tadzhikistan), China (China North-Central, China Southeast); Asia-Tropical: Indian Subcontinent (India), Indo-China (Myanmar, Vietnam); Northern America:	Lawns, roadsides, clearings, riparian habitats, parks, urban areas.<br>Alt. 0 – 1700 m	Duftveilchen, Common violet, English or sweet violet, Viola mammola, Violette odorante, Violeta común, Violeta de olor	[23,32,33,41,422

**Accepted name and protologue** [22]	**Family** [23]	**Synonyms**	**Lifeform** [24]	**Geographical distribution** [25]	**Habitat**	**Common names**	**References (and other literature therein)**
		ex Rchb., Fl. Germ. Excurs. 2: 705 (1832); *Viola sarmentosa* M.Bieb., Fl. Taur.-Caucas. 1: 172 (1808); *Viola spectabilis* K.Richt., Oesterr. Bot. Z. 35: 419 (1885); *Viola stevenii* Besser ex Fauc., Excurs. Bot. Valais: 96 (1872); *Viola suaveolens* Schur, Verh. Naturf. Vereins Brünn 15(2): 107 (1877); *Viola suavissima* Jord., Mém. Acad. Sci. Lyon, Sect. Sci. 1: 21 (1852); *Viola subcarnea* Jord., Mém. Acad. Sci. Lyon, Sect. Sci. 1: 17 (1852); *Viola sulfurea* Cariot, Étude Fl., éd. 7, 2: 81 (1884); *Viola vilmoriniana* Delacour & Mottet, Bull. Soc. Bot. France 46: 120 (1899); *Viola vinealis* Boreau, Fl. Centre France, ed. 3, 2: 75 (1857); *Viola wiedemannii* Boiss., Fl. Orient. 1: 457 (1867).		Western Canada (British Columbia), Eastern Canada (Nova Scotia Ontario, Québec), Northwestern America (Colorado, Idaho, Oregon, Washington), Northern-Central USA (Illinois, Wisconsin), Northeastern USA (Connecticut, Maine, Massachusetts, Michigan, Pennsylvania, New York, Ohio Rhode I.), Southwestern USA (California, Utah), Southeastern USA (North Carolina), Mexico (Mexico Central, Mexico Southwest); Southern America: Central America (Costa Rica, El Salvador, Panamá), Caribbean (Bermuda, Cuba, Dominican Republic, Haiti, Leeward Is., Puerto Rico, Trinidad-Tobago); Northern South America (Venezuela), Western South America (Colombia, Ecuador, Guatemala, Peru,), Southern South America (Argentina Northeast, Argentina Northwest, Argentina South, Chile Central).			
